# *Phaseolus vulgaris* STP13.1 is an H^+^‐coupled monosaccharide transporter, present in source leaves and seed coats, with higher substrate affinity at depolarized potentials

**DOI:** 10.1002/pld3.585

**Published:** 2024-04-22

**Authors:** Joseph L. Pegler, John W. Patrick, Benjamin McDermott, Anthony Brown, Jackson M. J. Oultram, Christopher P. L. Grof, John M. Ward

**Affiliations:** ^1^ Centre for Plant Science, School of Environmental and Life Sciences, College of Engineering, Science and Environment University of Newcastle Callaghan New South Wales Australia; ^2^ Plant and Microbial Biology University of Minnesota Twin Cities St. Paul Minnesota USA

**Keywords:** functional characterization, H^+^‐coupled hexose transport, *Phaseolus vulgaris*, PvSTP13.1

## Abstract

Sugar transport proteins (STPs) are high‐affinity H^+^‐coupled hexose symporters. Recently, the contribution of STP13 to bacterial and fungal pathogen resistance across multiple plant species has garnered significant interest. Quantitative PCR analysis of source leaves, developing embryos, and seed coats of *Phaseolus vulgaris* *L*. (common bean) revealed that *PvSTP13.1* was expressed in source leaves and seed coats throughout seed development. In contrast, *PvSTP13.1* transcripts were detected at exceedingly low levels in developing embryos. To characterize the transport mechanism, PvSTP13.1 was expressed in *Xenopus laevis* oocytes, and inward‐directed currents were analyzed using two‐electrode voltage clamping. PvSTP13.1 was shown to function as an H^+^‐coupled monosaccharide symporter exhibiting a unique high affinity for hexoses and aldopentoses at depolarized membrane potentials. Specifically, of the 31 assessed substrates, which included aldohexoses, deoxyhexoses, fructose, 3‐O‐methyl‐D‐glucose, aldopentoses, polyols, glycosides, disaccharides, trisaccharides, and glucuronic acid, PvSTP13.1 displayed the highest affinity (*K*_0.5_) for glucose (43 μM), mannose (92 μM), galactose (145 μM), fructose (224 μM), xylose (1.0 mM), and fucose (3.7 mM) at pH 5.6 at a depolarized membrane potential of −40 mV. The results presented here suggest PvSTP13.1 contributes to retrieval of hexoses from the apoplasmic space in source leaves and coats of developing seeds.

## 1 INTRODUCTION

Following fixation of atmospheric carbon dioxide (CO_2_) in photosynthetically active leaf mesophyll cells (source), excess sugars (primarily sucrose) are transported over long distances via sieve element/companion cell complexes within the phloem to heterotrophic (sink) organs. Loading and unloading of sucrose into, and from, the phloem generates an osmotically regulated turgor pressure difference, which ensures a continuous bulk flow of resources (nutrients, signaling molecules, sugars, and water) from source to sink (Jensen, 2018).

In those plant systems in which there is an apoplasmic step in phloem loading and/or unloading of sucrose, plasma membrane‐located transport proteins mediate movement between abutting symplasmic domains during sucrose passage from source to sink (Milne et al., 2018; Zhang & Turgeon, 2018). To date, members of three sucrose transport families have been identified that perform this function. These are H^+^‐coupled sucrose‐uptake transporters (SUTs), sugars will eventually be exported transporters (SWEETs; Milne et al., 2018; Zhang & Turgeon, 2018), and Sucrose and Glucose Carrier 1 (SUGCAR1; Yang et al., 2022). Furthermore, in some apoplasmic unloading pathways, released sucrose is hydrolyzed by cell wall invertases with the hexose products retrieved by plasma membrane‐located transport proteins. Here, the responsible plasma membrane transporters are sugar transport proteins (STPs), SWEETs, and SUGCAR1 (Milne et al., 2018; Yang et al., 2022).

Of the sugar transporter families expressed in sinks, the functional role of STPs is least understood. A circumstance possibly arises from the common characteristic of high numbers of STP isoforms being co‐expressed in a sink conferring redundancy for hexose transport (Pegler et al., 2023; Wen et al., 2022). Unlike SWEET uniporters, STPs are high affinity (apparent *K*_*m*_ 2 to 100 μm) hexose symporters that underpin a potential capacity to accumulate hexoses to high concentrations from apoplasmic spaces in post‐phloem unloading routes (Pegler et al., 2023; Pommerrenig et al., 2020; Wen et al., 2022). Thus far, this functional role has only been demonstrated for STPs expressed in storage cells of fleshy fruits that accumulate hexoses to high levels (Milne et al., 2018; Wen et al., 2022).

Based on the capacity to confer resistance to bacterial and fungal pathogens, foliar‐expressed STP13s have emerged as a hexose symporter of significant interest (Milne et al., 2023; Wen et al., 2022). Resistance would appear to be conferred in two ways. A variant of wheat STP13, a non‐functional hexose symporter encoded by the *Leaf rust resistance* (*Lr67res*) gene, confers resistance to fungal attack in wheat (Milne et al., 2019, 2023; Moore et al., 2015) and when heterologously expressed in *Medicago truncatula* (Gupta et al., 2021). This resistant phenotype is duplicated by the orthologous mutated *M. truncatula* MtSTP13.1^G144R^ (Gupta et al., 2021). In contrast, AtSTP13 and MtSTP13.1 function as hexose symporters and contribute to basal defense by outcompeting microbial pathogens for apoplasmic hexoses (Lemonnier et al., 2014; Yamada et al., 2016; Gupta et al., 2021). In relation to supporting growth, overexpression of AtSTP13 enhanced the growth of *Arabidopsis* seedlings (Schofield et al., 2009), whereas apple MdSTP13a has been shown to be essential for pollen tube elongation (Li et al., 2020). Unlike foliar‐expressed AtSTP13 (Pommerrenig et al., 2020), in addition to transporting hexoses, MdSTP13a also transports sucrose (Li et al., 2020); a characteristic shared with sugar beet BvSTP13 expressed in the sucrose accumulating taproots (Reyer et al., 2021).

Although members of many STP sub‐clades are expressed in developing seeds (Pegler et al., 2023), to date, STP13s have not been included in this cohort. In order to explore the role of STPs in seed filling, we investigated whether *STP13* was expressed in source leaves and recipient‐developing seeds (coat and embryo separately) across the pre‐ and storage phases of *Phaseolus vulgaris L*. (common bean). A qPCR analysis demonstrated that *PvSTP13.1* was measurably expressed in source leaves and seed coats but only to vanishingly low levels in embryos. This finding prompted a rigorous functional characterization of PvSTP13.1 by heterologous expression in *Xenopus laevis* oocytes to directly test for electrical coupling, sugar selectivity, and binding affinities. Our analysis indicated that PvSTP13.1 is a high‐affinity H^+^‐coupled monosaccharide symporter with a preference for hexoses and aldopentoses that showed high substrate affinity at depolarized membrane potentials. We discuss a potential role of PvSTP13.1 contributing to seed coat development and, in particular, during the pre‐storage phase of seed development.

## 2 MATERIALS AND METHODS

### 2.1 Phylogenetic analysis and amino acid sequence alignment

The amino acid sequence of each accession was retrieved from Phytozome v13 (https://phytozome-next.jgi.doe.gov/; Goodstein et al., 2012). Amino acid sequences were retrieved from either the *Arabidopsis thaliana* genome v11.0 (Cheng et al., 2017), *Malus domestica* v1.1 (Daccord et al., 2017), *M. truncatula* genome v4.0 (Tang et al., 2014), or *P. vulgaris* genome v2.1. Regarding sequences retrieved from the *P. vulgaris* genome v2.1, these sequence data were produced by the US Department of Energy Joint Genome Institute. The phylogenetic tree and amino acid sequence alignment were constructed in Geneious prime 2021.2.2.

### 2.2 Plant growth conditions

*P. vulgaris* (cv. Redland Pioneer) were cultivated in 2‐L pots containing Coir Peat, coarse river sand, and Perlite (1:1:1). Each pot received 15 g of Osmocote Exact Standard 8–9 months slow‐release fertilizer and thereafter fertilized once a week with either Peters Excel Cal Mag (1 g/L) or Wuxal Liquid Fertilizer (2 mL), with each being applied biweekly.

After germination, seedlings were thinned to one plant per pot and grown in a glasshouse at 24°C/14 h, 15°C/10 h day/night settings. For plants raised during winter months, the photoperiod was extended by supplemental lighting in the morning and afternoon to maintain a 14‐h day length. Each pot was watered with an automatic dripper system to maintain field capacity. Upon opening of the first flower bud, plants were transferred to growth cabinets with 24°C/14 h, 15°C/10 h day/night settings. During light hours, plants were chronologically illuminated with 3 h/200 μmol m^−2^ s^−1^ photosynthetic active radiation (PAR), 8 h/400 μmol m^−2^ s^−1^ PAR, and 3 h/200 μmol m^−2^ s^−1^ PAR.

Plants were de‐topped above trifoliate leaf 5 and pruned to remove axillary buds/branches at subtending nodes 1 and 5 to retain reproductive morphological units borne at nodes 2, 3, and 4 each supported by a trifoliate leaf. Flowers were tagged at anthesis (i.e., full petal emergence), and at 7 days thereafter, each axil branch was pruned to retain two developing pods supported by a trifoliate leaf.

### 2.3 RNA isolation and quantitative RT‐PCR

At specified days post anthesis (11, 16, 21, 26, and 31 DPA), pods were harvested and transferred to the laboratory on ice. These time points were selected as 11 to 21 DPA occur in the pre‐storage phase, whereas 21 to 31 DPA occur in the storage phase, with the latter DPA approaching cessation of seed fill (Thomas et al., 2000). Seed coats were surgically separated from embryos, weighed to ensure correct developmental stage, and thereafter immediately snap frozen in liquid nitrogen. Each of four biological replicates was composed of seed coats or embryos sampled from four pods harvested from randomized nodes of four independent plants. To isolate tissue from the source leaf tending the growing pods at 21 DPA, an 8‐mm cork borer removed two disks of lamina (avoiding secondary veins) adjacent to the midvein of each blade of the trifoliate leaf. Each of the four biological replicates was composed of six lamina disks from randomized nodes of four independent plants. Source leaves tending the growing pods at 21 DPA were selected as this corresponded with the highest expression of *PvSTP13.1* observed in seed coats. The RNA of each biological replicate was isolated using the Spectrum™ Plant Total RNA Kit (Sigma‐Aldrich, Australia), according to the manufacturer's instructions. Isolated RNA samples were DNase I (New England Biolabs, Australia) treated according to the manufacturer's instructions. Dnase‐treated RNA reactions were purified with the Monarch® RNA Cleanup Kit (New England Biolabs, Australia), according to the manufacturer's instructions. A total of 1.0 μg of RNA was used as a template for cDNA synthesis with 1.0 U of ProtoScript® II Reverse Transcriptase (New England Biolabs, Australia) according to the manufacturer's instructions along with 2.5 μM of oligo dT(18). All single‐stranded cDNA preparations were diluted to 50 ng/μL in Rnase‐free water prior to RT‐qPCR quantification of mRNA transcript abundance. The GoTaq® qPCR Master Mix (Promega, Australia) was used as the fluorescent reagent for all performed RT‐qPCRs, with the RT‐qPCRs cycling conditions set to 1 cycle of 95°C for 10 min, followed by 45 cycles of 95°C for 10 s and 60°C for 15 s. The relative abundance of the *PvSTP13.1* (Phvul.002G046800) and *PvSTP13.2* (Phvul.007G055100) mRNA transcripts were determined using the 2^−ΔΔCT^ method with *ALKBH6* (Phvul.004G131600), *HBP* (Phvul.004G094900), and *IDE* (Phvul.001G133200) acting as the internal controls. All DNA oligonucleotides used for the quantification of mRNA expression are provided in Table S2.

### 2.4 *Pv*STP13.1 complementary RNA synthesis for expression in *Xenopus laevis* oocytes

The coding sequence of PvSTP13.1 was optimized for *X. laevis* codon usage, synthesized with flanking gateway attL1 and attL2 recombination sites, and cloned into the vector “Blue Heron pUCKan” (Blue Heron Biotech). Using LR clonase recombinase according to manufacturer's instructions (Life Technologies), PvSTP13.1 was recombined into the destination vector, pOO2/GW (Sun et al., 2010). The plasmid was linearized with the restriction enzyme, *Mlu*I (New England Biolabs) and subsequently transcribed into complementary RNA using the Ambion mMessage mMachine kit according to the manufacturer's instructions (Life Technologies).

### 2.5 Functional characterization of *Pv*STP13.1 expressed in *Xenopus laevis* oocytes

Stage V and VI oocytes of *X. laevis* were isolated, processed, and injected with 50 nL (1.1 ng nL^−1^) of cRNA as described in Sivitz et al. (2005). Four to 6 days after cRNA injection, oocytes were submerged in a recording bath containing MT Ringer solution (MT Ringer; 115‐mM NaCl, 1‐mM KCl, 1.8‐mM CaCl_2_, 1‐mM CaCl_2_, and 5‐mM MES‐Tris at a pH of 5.6 or 6.8). For K^+^ Ringer, NaCl was replaced with KCl. For choline Ringer, NaCl and KCl were replaced with choline chloride. Glass recording pipettes filled with 1 M KCl and determined to have a resistance between 1 to 3 megaohms were used to measure currents by the two‐electrode voltage‐clamp technique (Sivitz et al., 2005). Currents were measured using a Dagan TEV200A amplifier (Dagan Corp., Minneapolis, MN) and digitized using pClamp 6 (Axon Instruments, Inc., Union City, CA).

## 3 RESULTS

### 3.1 The sugar transport protein (STP) family in *Phaseolus vulgaris*

**FIGURE 1:**
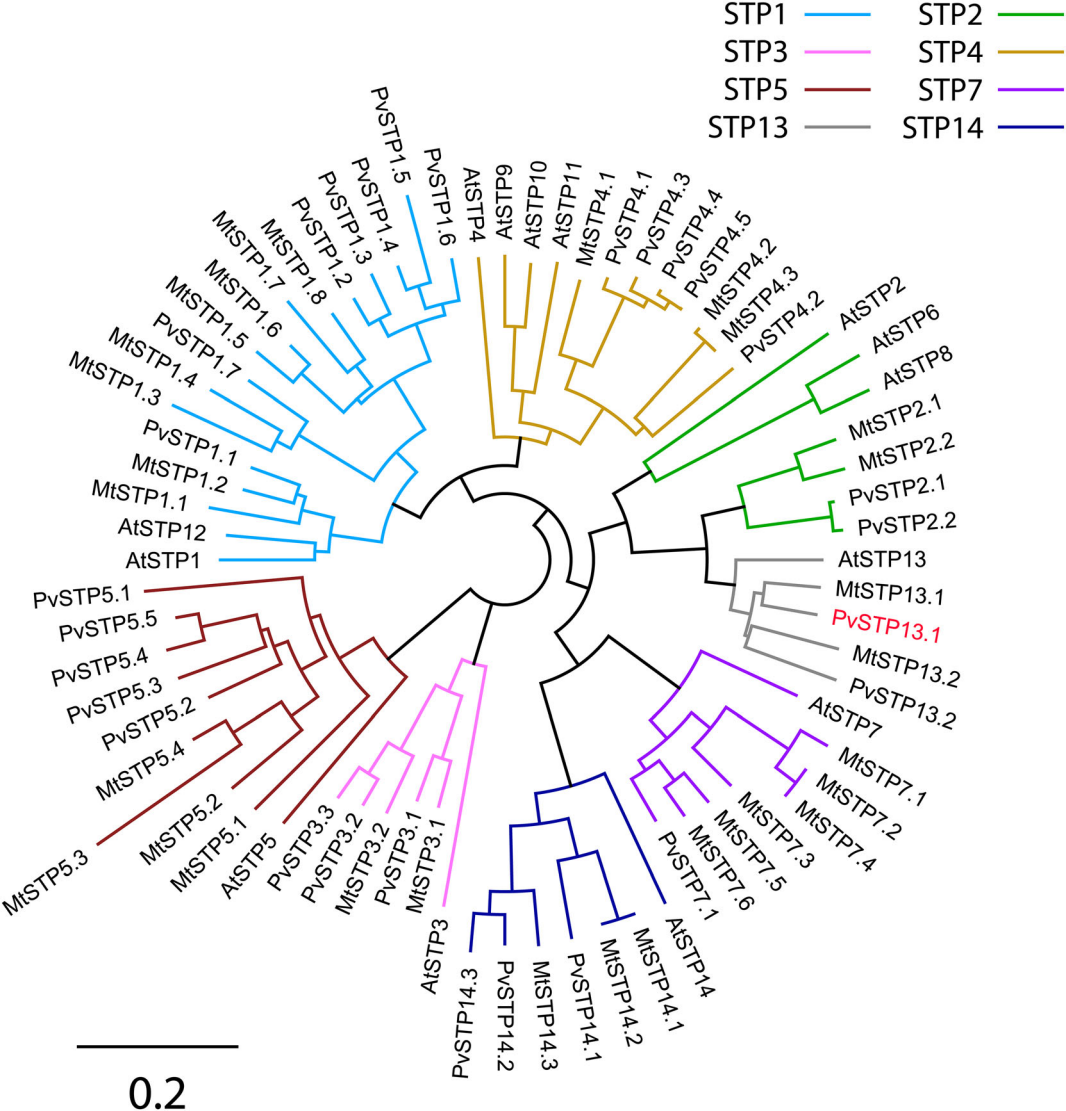
Phylogenetic analysis of PvSTP13.1. A total of 14, 30, and 28 STPs were retrieved from the *Arabidopsis thaliana* genome v11.0, *Medicago truncatula* genome v4.0, and *Phaseolus vulgaris* genome v2.1, respectively. Each STP is named according to the nomenclature proposed by Doidy et al. (2019), with the respective accession number provided in Table S1. The red text indicates PvSTP13.1.

**FIGURE 2:**
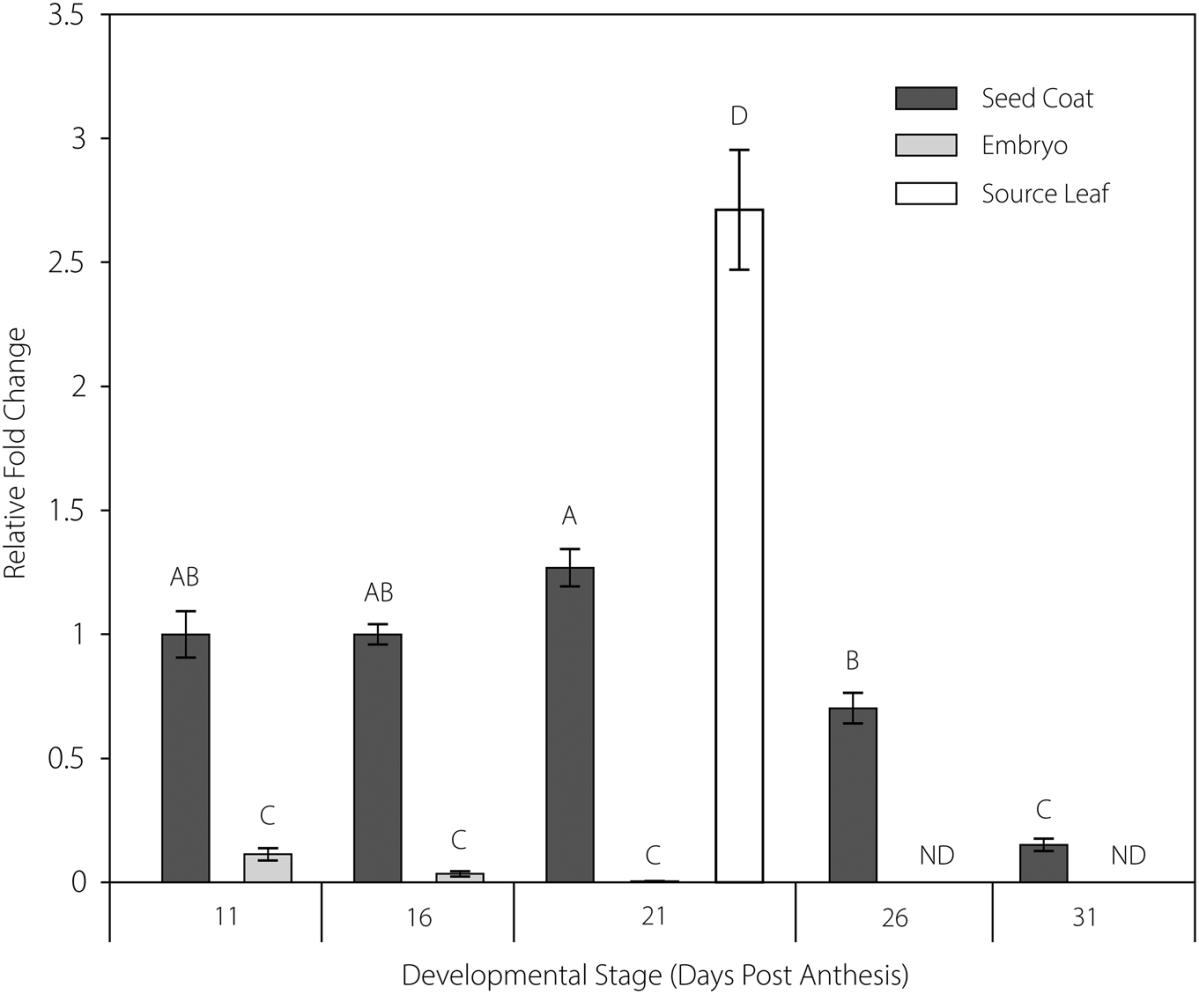
Quantitative PCR determination of (a) *PvSTP13.1* and (b) *PvSTP13.2* expression in seed coat and embryo at 11, 16, 21, 26, and 31 days post anthesis and the source leaf. Error bars represent the standard deviation of four biological replicates. One‐way ANOVA and Tukey's post hoc tests were used to determine statistically significant differences in expression. Statistically significant differences (*p* < .05) are indicated by different letters above the respective columns. “ND” indicates that the transcript was not detected.

Using sequences of previously identified STPs from *Arabidopsis* and *M. truncatula*, 28 STPs were identified in the genome of *P. vulgaris* (Figure 1). Categorized into the eight subclades (STP1, STP2, STP3, STP4, STP5, STP7, STP13, STP14), a total of 7, 2, 3, 5, 5, 1, 2, and 3 STPs grouped into each, respectively. Each of the identified PvSTPs was named according to the revised nomenclature proposed by Doidy et al. (2019). This analysis identified two *P. vulgaris* STP13 isoforms, PvSTP13.1 and PvSTP13.2, which are 92% similar at the amino acid level (Figure S1). PvSTP13.1 was selected as the focus of this study as qPCR analysis found its transcript to be expressed in the source leaf and in the seed coats throughout seed development (11–31 DPA), with expression peaking at 21 DPA (Figure 2). Compared with seed coats at 11 DPA, *PvSTP13.1* expression was increased by 1.27‐ and 2.71‐fold at 21 DPA in seed coats and source leaves tending the growing pods, respectively. In contrast, *PvSTP13.1* was found to be expressed at very low levels in the embryo at 11, 16, and 21 DPA, and expression was not detectable thereafter. The other *PvSTP13* isoform, *PvSTP13.2*, was not investigated further because of its very low expression in all examined tissues compared with *PvSTP13.1* (Table S3).

### 3.2 PvSTP13.1 functions as a hexose/proton symporter

**FIGURE 3:**
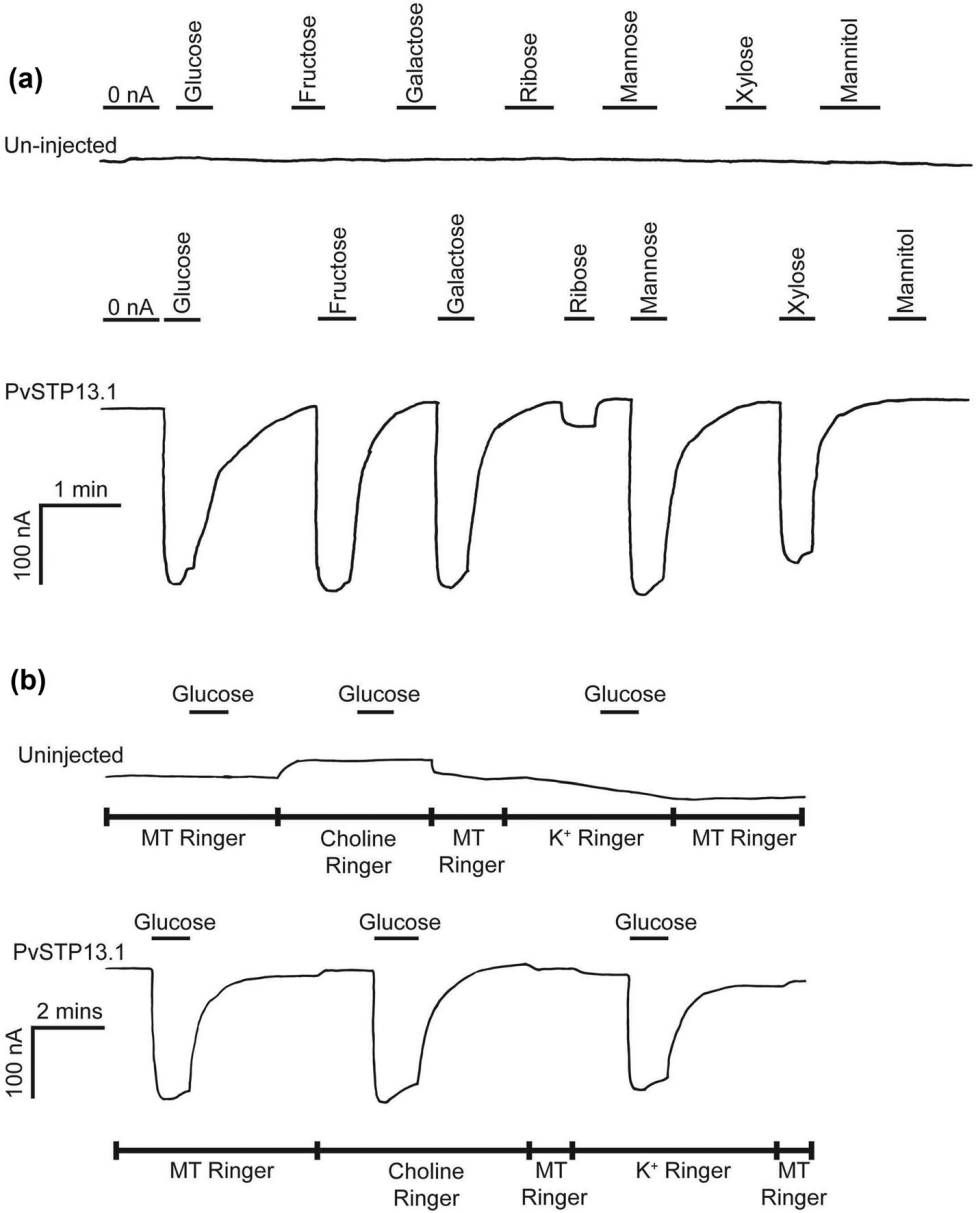
Current trace of *Xenopus laevis* oocytes either non‐injected or expressing PvSTP13.1. (a) Oocytes either non‐injected or injected with PvSTP13.1 cRNA were bathed in MT Ringer solution (pH 5.6) containing glucose, fructose, galactose, ribose, mannose, xylose, or mannitol, with each substrate supplied at a concentration of 10 mM. (b) Current traces of single oocytes either non‐injected or injected with PvSTP13.1 cRNA and supplied with 10‐mM glucose in MT Ringer, choline Ringer, or K^+^ Ringer. Each oocyte was voltage‐clamped at −40 mV. The line presented under each sugar label is indicative of its addition and removal from the oocyte bathing solution. The scale in the bottom left corner of panels (a) and (b) applies to the non‐injected and PvSTP13.1 injected oocytes presented.

The substrate specificity of PvSTP13.1‐expressing oocytes was investigated using the two‐electrode voltage clamp technique. With the membrane potential set to −40 mV, each substrate was supplied to oocytes at a 10‐mM concentration in MT Ringer solution buffered at pH 5.6. This millimolar substrate concentration exceeds the micromolar *K*_m_ range reported for STP13 homologs (Li et al., 2020; Milne et al., 2023; Nørholm et al., 2006; Reyer et al., 2021), ensuring the optimal detection of any proton‐coupled sugar transport, as PvSTP13.1 would theoretically be functioning at *V*_max_. Large inward‐directed currents were induced in response to the presence of hexoses, glucose, fructose, galactose, mannose, and the aldopentose, xylose (Figure 3a). When supplied with the aldopentose ribose, a small inward directed current was observed, whereas no change in current occurred in response to the presence of the polyol, mannitol. To support the possibility that inward‐induced currents were a result of a proton‐coupled mechanism of sugar transport, 10‐mM glucose was supplied to both non‐injected and oocytes expressing PvSTP13.1 in choline Ringer and K^+^ Ringer solutions buffered at pH 5.6 (Figure 3b). The retention of large glucose‐induced inward currents strongly indicated that PvSTP13.1 transport of sugars is proton‐coupled and not dependent on Na^+^ or K^+^ present in the MT Ringer solution (Figure 3b). None of the sugars supplied to non‐injected oocytes induced a notable change in current (Figure 3a–b).

### 3.3 Substrate specificity of PvSTP13.1

**FIGURE 4:**
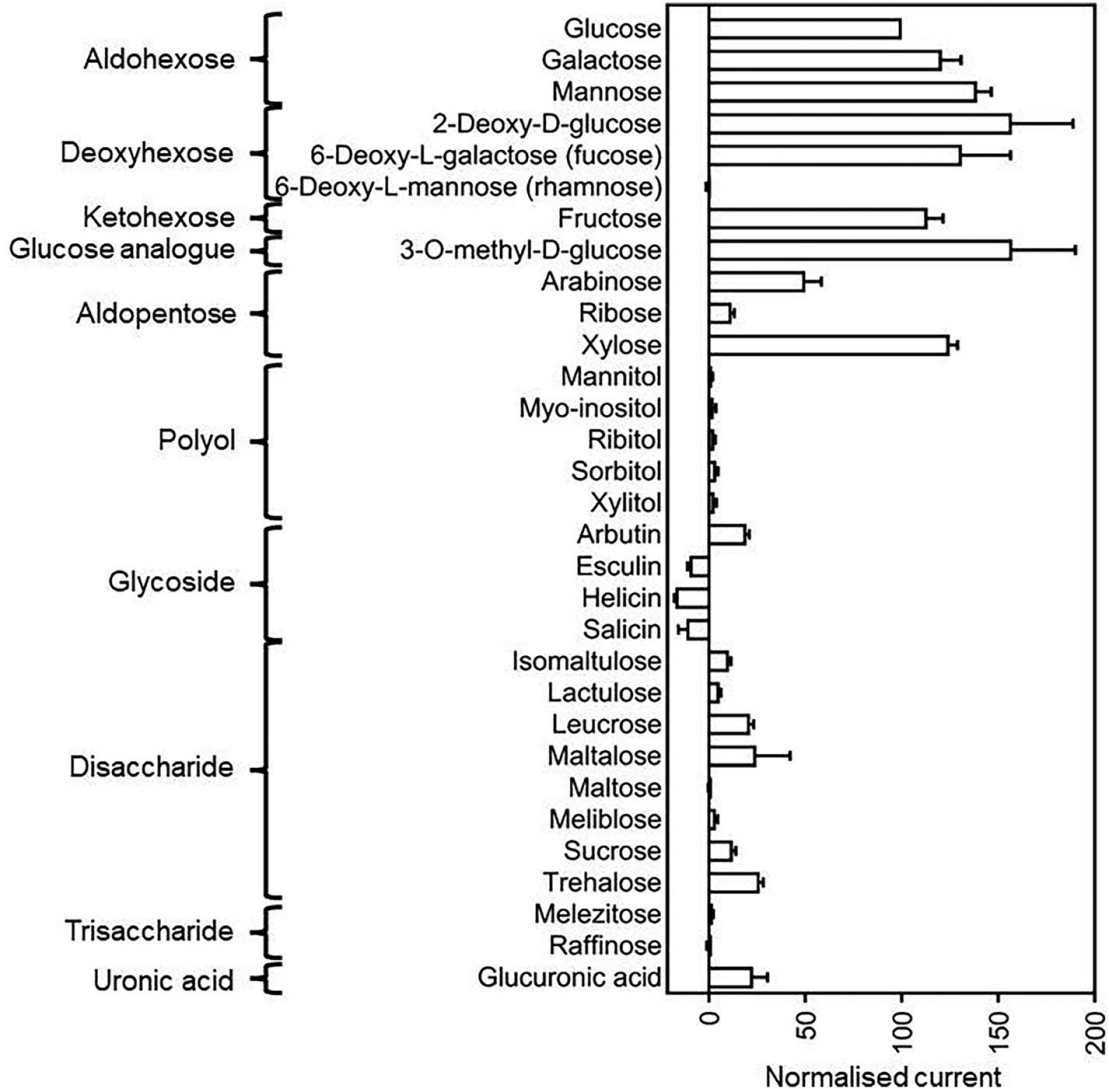
Substrate specificity of PvSTP13.1. Substrate‐dependent currents were measured at a membrane potential of −137.6 mV. Each substrate was supplied at a concentration of 10 mM in MT Ringer solution (pH 5.6) except for esculin, which was supplied at a concentration of 2.5 mM due to its solubility limitation. Substrate currents were normalized to the currents obtained for 10‐mM glucose with the same oocyte. The data presented are the mean ± SEM of three oocytes.

A wide range of potential substrates was tested for their ability to induce inward currents. An additional 24 substrates were supplied at a concentration of 10 mM (or 2.5 mM in the case of esculin) to oocytes expressing PvSTP13.1 (Figure 4). Voltage pulses from −137.6 to 57.5 mV were applied to oocytes clamped at a holding potential of −40 mV. The average (*n* = 3) inward‐directed currents induced at −137.6 mV are shown in Figure 4 relative to the currents recorded for glucose. The exposure to aldohexoses (galactose, glucose, and mannose), deoxyhexoses (2‐deoxy‐d‐glucose and 6‐deoxy‐L‐galactose [fucose]), the ketohexose, fructose, and the glucose analog 3‐O‐methyl‐D‐glucose each resulted in strong inward currents. The presence of deoxyhexose, 6‐deoxy‐L‐mannose (rhamnose), had no impact on the currents measured for PvSTP13.1‐expressing oocytes. The application of aldopentoses induced varied levels of inward‐directed current. Specifically, arabinose induced a smaller inward‐directed current of 50.0%, in comparison with glucose. In contrast, application of xylose resulted in a large inward‐directed current 124.8% of that recorded for glucose. Application of the aldopentose, ribose, select polyols (mannitol, myo‐inositol, ribitol, sorbitol, and xylitol), glycosides (arbutin, esculin, helicin, and salicin), disaccharides (maltose, isomaltulose, lactulose, leucrose, maltalose, meliblose, sucrose, and trehalose), trisaccharides (melezitose and raffinose), and glucuronic acid each resulted in very small current changes that were attributed to “background.”

### 3.4 Kinetic analysis of glucose transport by PvSTP13.1

**FIGURE 5:**
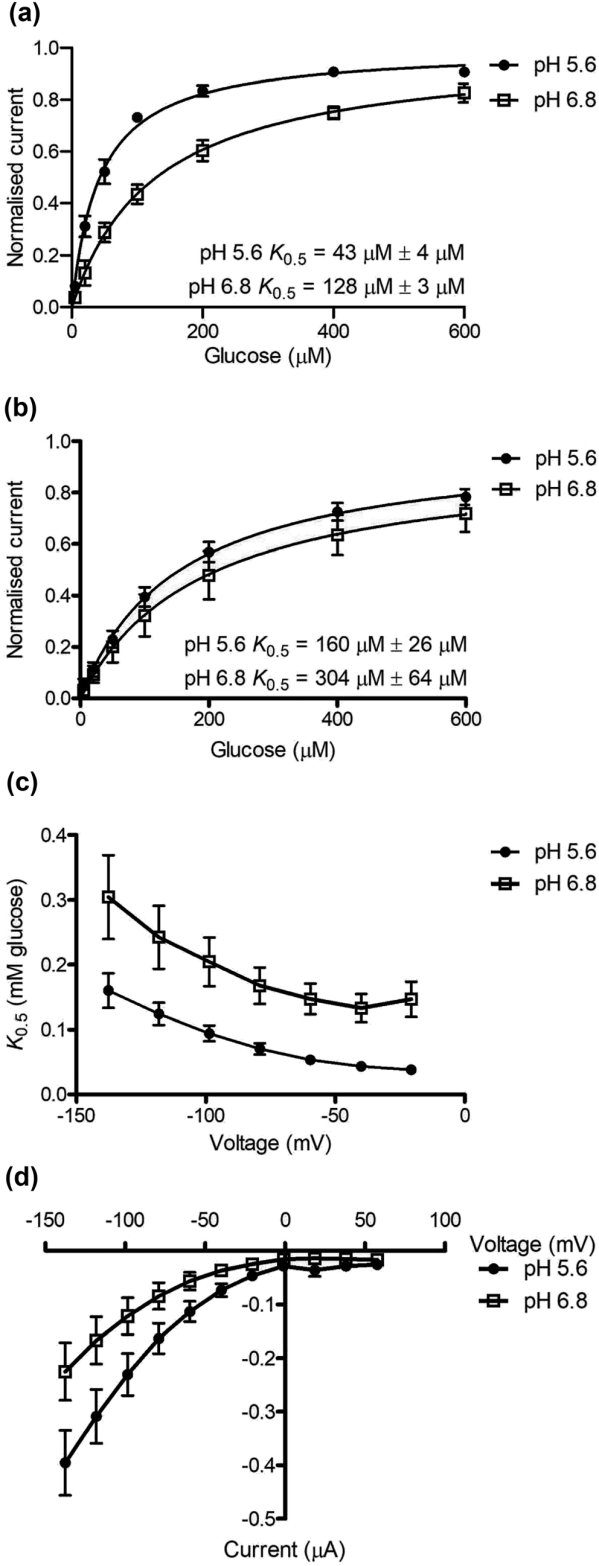
Concentration and voltage dependence of PvSTP13.1 affinity for glucose at pH 5.6 and 6.8. PvSTP13.1 was expressed in *Xenopus laevis* oocytes, and recordings were measured using the two‐electrode voltage clamping technique. Concentration‐dependent transport with currents recorded at a membrane potential of (a) −40 mV and (b) −137.6 mV were fitted with a Michaelis–Menten curve, which was then normalized to *V*_max_ due to the variation in *V*_max_ between individual oocytes, (c) substrate affinity in response to voltage, and (d) current–voltage relationship for glucose‐dependent currents at pH 5.6 and 6.8. Substrate‐dependent currents were determined by subtracting the average current recorded prior to, and following, glucose addition from the current measured during the presence of 10‐mM glucose. All data presented are the mean ± SEM of three oocytes.

The voltage and pH dependence of the affinity of PvSTP13.1 for glucose was assessed. As membrane potentials of seed coats have been shown to range from −40 to −55 mV in vivo (Van Dongen et al., 2003; Walker et al., 1995), the affinity (*K*_0.5_) of PvSTP13.1 for glucose was measured at a membrane potential of −40 mV (Figure 5a) and −137.6 mV (Figure 5b) at pH 5.6 and 6.8. Using seven glucose concentrations between 5 and 600 μM, at a membrane potential of −40 mV, the *K*_0.5_ of PvSTP13.1 for glucose was determined to be 43 μM at pH 5.6 (Figure 5a). At alkaline conditions of pH 6.8, the *K*_0.5_ tripled, indicating that the affinity for glucose is strongly pH dependent. This pH dependence was similarly observed at a membrane potential of −137.6 mV with the *K*_0.5_ (160 μM) of PvSTP13.1 for glucose at pH 5.6 approximately double that at pH 6.8 (Figure 5b). The four‐fold reduction in *K*_0.5_ of PvSTP13.1 for glucose at −137.6 mV compared with *K*_0.5_ at −40 mV indicates that PvSTP13.1 functions as a very high‐affinity transporter at depolarized potentials. At pH 5.6 and 6.8, the affinity of PvSTP13.1 increased as the membrane potential was increasingly depolarized from −137.6 to −20.6 mV (Figure 5c). The glucose‐dependent currents were not voltage dependent in the range of −40 to −137.6 mV at pH 5.6 or 6.8 and did not reverse at positive potentials (Figure 5d).

### 3.5 Kinetic analysis of PvSTP13.1‐facilitated transport of other monosaccharides

**FIGURE 6:**
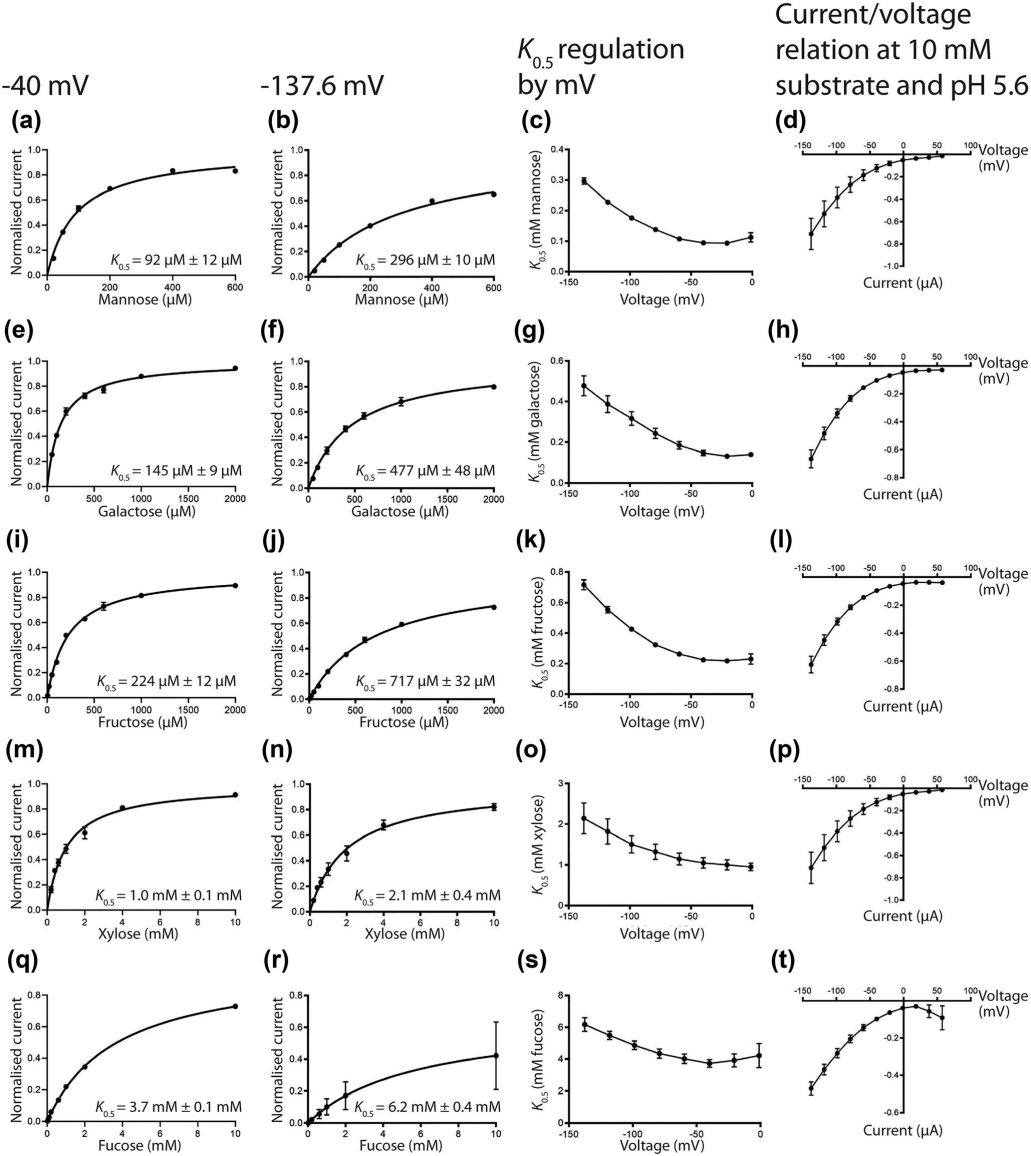
Concentration and voltage dependence of PvSTP13.1 affinity for monosaccharides. PvSTP13.1 was expressed in *Xenopus laevis* oocytes, and recordings were measured using the two‐electrode voltage clamping technique. Concentration‐dependent transport, substrate affinity in response to voltage, and the current versus voltage relationship determined for mannose (a–d), galactose (e–h), fructose (i–l), xylose (m–p), and fucose (q‐t) at pH 5.6. All currents are substrate‐dependent (background subtracted) and presented as mean ± SEM of three oocytes. To assess concentration‐dependent transport, currents recorded at −40 mV (a, e, i, m, q) or −137.6 mV (b, f, j, n, r) were fitted with a Michaelis–Menten curve, which was then normalized to *V*_max_ due to the variation in *V*_max_ between individual oocytes. The current–voltage relationship of PvSTP13.1 expressing oocytes was determined at 10‐mM substrate concentration.

Because of the large inward‐directed currents observed for the monosaccharides, fructose, fucose, galactose, mannose, and xylose (Figure 4), concentration and voltage dependence of PvSTP13.1 for these sugars was evaluated. At pH 5.6 and a depolarized membrane potential of −40 mV, the *K*_0.5_ of PvSTP13.1 for aldohexoses, mannose (92 μM) (Figure 6a) and galactose (145 μM) (Figure 6e), were approximately two and three times larger than the *K*_0.5_ determined for glucose (43 μM). A further reduction in substrate affinity was observed following exposure of PvSTP13.1 expressing oocytes to the ketohexose, fructose with a *K*_0.5_ approximately five times larger (224 μM) than that observed for glucose (Figure 6I). The *K*_0.5_ values of PvSTP13.1 for the aldopentose, xylose (Figure 6M) and deoxyhexose, fucose (Figure 6Q) were an order of magnitude higher than those observed for mannose, galactose, and fructose, requiring mM concentration of each substrate. The *K*_0.5_ of PvSTP13.1 for each of these substrates was two to four times higher when measured at a membrane potential of −137.6 mV (Figure 6b,f,j,n,r). As for glucose, the affinity of PvSTP13.1 for each of the assessed monosaccharides increased as the membrane potential became increasingly depolarized, plateauing at −40 mV (Figure 6c,g,k,o,s). Consistent with the current–voltage relationship for glucose, all tested substrates produced currents that were not voltage dependent and did not reverse (Figure 6d,h,l,p,t).

## 4 DISCUSSION

Phylogenetic analysis of the common bean genome identified the presence of 28 STPs belonging to the eight STP clades (Figure 1). Of the 28 identified STPs, notably, *PvSTP13.1* was determined to be present in the source leaf and seed coat throughout seed development but was expressed at low levels in embryos until 21 DPA and thereafter was non‐detectable (Figure 2). In a recent study, *PvSTP13.1* transcript was found to be present in seedling, shoot, and root tissues (Shalimar French Bean‐1 variety) with expression induced in both tissues in response to mineral deficiency (Urwat et al., 2021).

Electrophysiological studies across diverse plant species have demonstrated the capacity of STP13 to undertake proton‐coupled symport of hexoses with the highest affinity for glucose (Milne et al., 2023; Nørholm et al., 2006; Reyer et al., 2021). This proton‐coupled symport of monosaccharides was similarly observed for PvSTP13.1 (Figure 3a–b). Specifically, the heterologous expression of PvSTP13.1 in *X. laevis* oocytes resulted in large inward currents when exposed to MT Ringer solution supplemented with 10‐mM concentrations of monosaccharides, glucose, fructose, fucose, galactose, mannose, and xylose (Figures 3a and 4). The preference of STP13 for a broad range of hexoses has been observed in wheat, *Arabidopsis*, apple, and sugar beet, with a demonstrated strong selectivity for the aldopentose xylose, observed in the latter three species (Li et al., 2020; Moore et al., 2015; Nørholm et al., 2006; Reyer et al., 2021). Interestingly, MdSTP13a has a higher affinity for sucrose (*K*_m_ value = 66.9 μM) than glucose, and application of sucrose to BvSTP13 expressing oocytes results in inward‐directed currents ~60% of that observed for glucose (Li et al., 2020; Reyer et al., 2021). This high affinity for sucrose was not observed for PvSTP13.1, suggesting the amino acids present in the substrate binding site of PvSTP13.1 are less accommodating of the fructose moiety of sucrose in comparison to MdSTP13a and BvSTP13 (Figure S1; Paulsen et al., 2019; Li et al., 2020; Bavnhøj et al., 2021; Reyer et al., 2021). As the crystal structure of the known high affinity, proton‐coupled glucose symporter, AtSTP10 has been determined, the amino acid sequence of this protein was utilized to determine any residue substitutions that may contribute to the broad sugar specificity observed across STP13 (Figure S1). Except for Leu43 (replaced by valine), the amino acids central to coordinating glucose binding are conserved between AtSTP10 and the STP13 homologs. Recent 3D modeling of BvSTP13 bound with sucrose implicated the change of Asn304 and Met307 as necessary for sucrose binding (Reyer et al., 2021). As these amino acid residues are conserved in PvSTP13.1, further analysis is required to determine why there is variation in sucrose binding capability between STP13 homologs.

To assess the kinetic properties of PvSTP13.1, substrate concentration and membrane potential were manipulated to determine how each component contributed to PvSTP13.1 transport activity. At a clamped membrane potential of −40 and −137.6 mV, the *K*_0.5_ of PvSTP13.1 for glucose was 43 and 160 μM respectively at pH 5.6 (Figure 5a–b), values which are comparable with the glucose affinity determined for AtSTP13 (74 μM at pH 5.0; Nørholm et al., 2006), BvSTP13 (75 μM at pH 5.5; Reyer et al., 2021), TaSTP13/Lr67sus (110 μM at pH 5.0; Milne et al., 2023), and MdSTP13a (157 μM at pH 6.0; Li et al., 2020). In response to an elevated pH of 6.8, the affinity of PvSTP13.1 for glucose was reduced by approximately two‐ to three‐fold, indicating that PvSTP13.1 transport is pH dependent (Figure 5a–b). It was expected that PvSTP13.1 would function most efficiently at pH 5.6, as the extracellular fluid surrounding common bean seed coats is approximately pH 5.7 (Van Bel & Patrick, 1985). Further, for AtSTP13, HvSTP13, TaSTP13/Lr67sus, and MdSTP13a, pH optima have been determined to be approximately 5.5 to 6.0, with exposure to basic pH conditions ≥7.0 significantly compromising the transport capacity of STP13 (Li et al., 2020; Milne et al., 2019; Moore et al., 2015; Nørholm et al., 2006).

When membrane potential is modulated, the affinity of PvSTP13.1 for glucose (Figure 5a–c), mannose (Figure 6a–c), galactose (Figure 6e–g), fructose (Figure 6i–k), xylose (Figure 6m–o), and fucose (Figure 6q–s) increased in response to depolarizing conditions, reaching its highest affinity and plateauing at membrane potentials of approximately −40 mV and above. This voltage‐dependent trend of PvSTP13.1 affinity for its transported substrates has also been observed for STP13 transport of glucose in sugar beet (Reyer et al., 2021) and wheat (Milne et al., 2023). Interestingly, this increase in affinity in response to depolarizing conditions is opposite to what has been observed for SUTs (Milne et al., 2017; Sivitz et al., 2005). This finding indicates that PvSTP13.1 transport activity is optimized to the *in planta* physiological conditions as the membrane potential of seed coats from common bean and pea have previously been shown to range from −40 to −55 mV when assessed in vivo (Walker et al., 1995; Van Dongen et al., 2003).

As reported for proton‐coupled sugar transporters (Sivitz et al., 2005, 2007) under depolarized conditions, current reversal is not observed for PvSTP13.1 (Figures 5d and 6d,h,l,p,t). This is partly due to a lack of substrate in the cytoplasm of the oocyte that would allow for sugar and proton efflux. The current/voltage relationship for PvSTP13.1 is linear between −40 and −137.6 mV for all transported substrates, indicating that in this range, the membrane potential is not regulating the rate of transport. This observation supports the work by Walker et al. (1995)) who concluded that net sucrose efflux from detached common bean seed coats was more heavily modulated by external and cytoplasmic pH than the membrane potential difference. However, at potentials more positive than −40 mV, currents approach zero for all substrates. This is an indication that the transporter is inwardly rectified, that is, the transport activity is downregulated at very depolarized potentials to prevent sugar efflux under depolarized conditions.

The electrophysiological data presented here identify PvSTP13.1 as a high affinity, proton‐coupled symporter that transports a broad range of monosaccharides, with a preference for hexoses and aldopentoses. This functional characterization of PvSTP13.1, in conjunction with qPCR determination of *PvSTP13.1* expression in the source leaves, and seed coat throughout seed development (Figure 2), strongly suggests that PvSTP13.1 retrieves hexoses from the leaf‐ and seed coat‐apoplasm. Recently, in *Arabidopsis*, STP13 has been shown to be highly and preferentially expressed in leaf guard cells where it is implicated as a potential key player in guard cell regulation during stress conditions (Flütsch et al., 2020). The temporal expression pattern of *PvSTP13.1* suggests that it is most active during the pre‐storage phase of seed development, a phase dominated by seed coat development and high cell wall invertase activity (Pegler et al., 2023). During the pre‐storage phase of Fava bean seed development, a high apoplasmic hexose/sucrose ratio is created by extracellular invertase activity (Weber et al., 1995). Unlike other STPs that have been shown to be expressed in both the maternal and filial tissues (Pegler et al., 2023), PvSTP13.1 appears to have a seed coat‐specific role in common bean. Specifically, PvSTP13.1 may play a significant role in maternal/filial competition for hexoses during the pre‐storage phase to preferentially support coat development.

## AUTHOR CONTRIBUTIONS

Conceptualization: Joseph L. Pegler, John W. Patrick, John M. Ward, Christopher P. L. Grof. Methodology: Joseph L. Pegler, John W. Patrick, John M. Ward, Christopher P. L. Grof. Investigation: Joseph L. Pegler, John M. Ward, Benjamin McDermott, Anthony Brown, Jackson M. J. Oultram. Analysis: Joseph L. Pegler, John M. Ward. Writing—original draft preparation: Joseph L. Pegler. Writing—review and editing: Joseph L. Pegler, John W. Patrick, John M. Ward, Christopher P. L. Grof. All authors have read and agreed to the published version of the manuscript.

## CONFLICT OF INTEREST STATEMENT

The authors have no conflicts of interest to declare.

## PEER REVIEW

The peer review history for this article is available in the Supporting Information for this article.

## Supporting information


**Data S1** Supporting Information

**Table S1:** The gene identifier of each gene used in the phylogenetic analysis presented in Figure 1.
**Table S2: DNA oligonucleotide sequences of quantitative PCR primers.** The reference genes, *ALKBH6* (Phvul.004G131600)*, HBP* (Phvul.004G094900) *and IDE* (Phvul.001G133200) were used to normalise the expression of *PvSTP13.1* (Phvul.002G046800) and *PvSTP13.2* (Phvul.007G055100).
**Table S2: DNA oligonucleotide sequences of quantitative PCR primers.** The reference genes, *ALKBH6* (Phvul.004G131600)*, HBP* (Phvul.004G094900) *and IDE* (Phvul.001G133200) were used to normalise the expression of *PvSTP13.1* (Phvul.002G046800) and *PvSTP13.2* (Phvul.007G055100).
**Table S3: Differential expression of *PvSTP13.1* and *PvSTP13.2* in the seed coat, embryo and source leaf of *Phaseolus vulgaris* .** These data are the mean of four biological replicates with two technical replicates. The data is presented as the mean ± standard error.
**Figure S1:** Amino acid sequence alignment of AtSTP10 (At3g19940), AtSTP13 (At5G26340) BvSTP13 (Bevul.3G139500), MdSTP13a (MD13G1189100), MtSTP13.1 (Medtr5g006070), MtSTP13.2 (Medtr1g104780), PvSTP13.1 (Phvul.002G046800), PvSTP13.2 (Phvul.007G055100) and TaSTP13 (Traes_4DL_CFC191A06). Amino acid residues encased in a red box indicate residues participating in AtSTP10 binding of sugar (Phe39, Leu43, Gln177, Ile184, Gln295, Gln296, Asn301, Asn332, Phe401, Gly406, Trp410, Asn433, Thr437; Paulsen et al., 2019). Amino acid residues encased in a blue box indicate the proton donor‐acceptor pair (Asp42 and Arg142) essential for AtSTP10 transport capability (Paulsen et al., 2019). Amino acid residues encased in a green box indicate the tri‐aromatic motif required for endoplasmic reticulum export and plasma membrane localisation of STPs (Yamada et al., 2017).

## Data Availability

The data underlying this article are available in the article and in its online supplementary material.
