# Supplementary material for: Phaseolus vulgaris STP13.1 is an H+‐coupled monosaccharide transporter, present in source leaves and seed coats, with higher substrate affinity at depolarized potentials
Source: Plant Direct. 2024 Apr 22;8(4):e585. doi: 10.1002/pld3.585 (PMC11033725; doi:10.1002/pld3.585)
Supplement: Supplementary file 2 — Table S1: The gene identifier of each gene used in the phylogenetic analysis presented in Figure 1. Table S2: DNA oligonucleotide sequences of quantitative PCR primers. The reference genes, ALKBH6 (Phvul.004G131600), HBP (Phvul.004G094900) and IDE (Phvul.001G133200) were used to normalise the expression of PvSTP13.1 (Phvul.002G046800) and PvSTP13.2 (Phvul.007G055100). Table S2: DNA oligonucleotide sequences of quantitative PCR primers. The reference genes, ALKBH6 (Phvul.004G131600), HBP (Phvul.004G094900) and IDE (Phvul.001G133200) were used to normalise the expression of PvSTP13.1 (Phvul.002G046800) and PvSTP13.2 (Phvul.007G055100). Table S3: Differential expression of PvSTP13.1 and PvSTP13.2 in the seed coat, embryo and source leaf of Phaseolus vulgaris . These data are the mean of four biological replicates with two technical replicates. The data is presented as the mean ± standard error. Figure S1: Amino acid sequence alignment of AtSTP10 (At3g19940), AtSTP13 (At5G26340) BvSTP13 (Bevul.3G139500), MdSTP13a (MD13G1189100), MtSTP13.1 (Medtr5g006070), MtSTP13.2 (Medtr1g104780), PvSTP13.1 (Phvul.002G046800), PvSTP13.2 (Phvul.007G055100) and TaSTP13 (Traes_4DL_CFC191A06). Amino acid residues encased in a red box indicate residues participating in AtSTP10 binding of sugar (Phe39, Leu43, Gln177, Ile184, Gln295, Gln296, Asn301, Asn332, Phe401, Gly406, Trp410, Asn433, Thr437; Paulsen et al., 2019). Amino acid residues encased in a blue box indicate the proton donor‐acceptor pair (Asp42 and Arg142) essential for AtSTP10 transport capability (Paulsen et al., 2019). Amino acid residues encased in a green box indicate the tri‐aromatic motif required for endoplasmic reticulum export and plasma membrane localisation of STPs (Yamada et al., 2017). [file PLD3-8-e585-s001.pdf]

Supplementary Table 1: The gene identifier of each gene used in the phylogenetic analysis presented in Figure 1.

**Plant species**	**Gene name**	**Gene identifier**
Arabidopsis thaliana	*AtSTP1*	At1g11260
	AtSTP2	At1g07340
	AtSTP3	At5g61520
	AtSTP4	At3g19930
	AtSTP5	At1g34580
	AtSTP6	At3g05960
	AtSTP7	At4g02050
	AtSTP8	At5g26250
	AtSTP9	At1g50310
	AtSTP10	At3g19940
	AtSTP11	At5g23270
	AtSTP12	At4g21480
	AtSTP13	At5G26340
	AtSTP14	At1g77210
Medicago truncatula	*MtSTP1.1*	Medtr4g090600
	MtSTP1.2	Medtr4g091370
	MtSTP1.3	Medtr3g023480
	MtSTP1.4	Medtr0009s0370
	MtSTP1.5	Medtr3g007910
	MtSTP1.6	Medtr3g093010
	MtSTP1.7	Medtr3g093060
	MtSTP1.8	Medtr5g041550
	MtSTP2.1	Medtr1g104750
	MtSTP2.2	Medtr1g104770
	MtSTP3.1	Medtr3g008160
	MtSTP3.2	Medtr3g008170
	MtSTP4.1	Medtr5g082540
	MtSTP4.2	Medtr1g038600
	MtSTP4.3	Medtr1g038630
	MtSTP5.1	Medtr3g074270
	MtSTP5.2	Medtr3g074390
	MtSTP5.3	Medtr3g074400
	MtSTP5.4	Medtr3g074410
	MtSTP7.1	Medtr4g116790
	MtSTP7.2	Medtr4g116770
	MtSTP7.3	Medtr0204s0020
	MtSTP7.4	Medtr0204s0040
	MtSTP7.5	Medtr5g094760
	MtSTP7.6	Medtr7g091690
	MtSTP13.1	Medtr5g006070
	MtSTP13.2	Medtr1g104780
	MtSTP14.1	Medtr6g087040
	MtSTP14.2	Medtr6g088375
	MtSTP14.3	Medtr8g102860
Phaseolus vulgaris	*PvSTP1.1*	Phvul.002G221200
	PvSTP1.2	Phvul.010G030300
	PvSTP1.3	Phvul.010G030000
	PvSTP1.4	Phvul.010G053400
	PvSTP1.5	Phvul.006G164200
	PvSTP1.6	Phvul.006G160700
	PvSTP1.7	Phvul.003G036800
	PvSTP2.1	Phvul.007G055500
	PvSTP2.2	Phvul.007G055200
	PvSTP3.1	Phvul.005G009500

	PvSTP3.2	Phvul.005G009400
	PvSTP3.3	Phvul.005G009300
	PvSTP4.1	Phvul.007G015600
	PvSTP4.2	Phvul.003G003400
	PvSTP4.3	Phvul.007G015400
	PvSTP4.4	Phvul.006G024300
	PvSTP4.5	Phvul.006G025166
	PvSTP5.1	Phvul.008G173200
	PvSTP5.2	Phvul.009G030700
	PvSTP5.3	Phvul.009G030800
	PvSTP5.4	Phvul.009G030500
	PvSTP5.5	Phvul.009G030600
	PvSTP7.1	Phvul.009G208801
	PvSTP13.1	Phvul.002G046800
	PvSTP13.2	Phvul.007G055100
	PvSTP14.1	Phvul.004G169600
	PvSTP14.2	Phvul.002G310500
	PvSTP14.3	Phvul.002G310600

Supplementary Table 2: DNA oligonucleotide sequences of quantitative PCR primers. The reference genes, *ALKBH6* (Phvul.004G131600), *HBP* (Phvul.004G094900) *and IDE* (Phvul.001G133200) were used to normalise the expression of *PvSTP13.1* (Phvul.002G046800) and *PvSTP13.2* (Phvul.007G055100).

**Primer name**	**Sequence (5' to 3')**	**Targeted sequence ID**	**Amplicon size**
PvALKBH6 FP	ATCCGCGCTATTCCCATCAC	Phvul.004G131600	140bp
PvALKBH6 RP	CCATGACAACAGGAGACCCA		
PvHBP FP	GGGAGAGTCTTCAGTCAGCG	Phvul.004G094900	131bp
PvHBP RP	CCACCTCAGCAAGGGCAATA		
PvIDE FP	GCAACCAACCTTTCATCAGC	Phvul.001G133200	156bp
PvIDE RP	AGAAATGCCTCAACCCTTTG		
PvSTP13.1 FP	CGCAGGAACAGACCACAGAT	Phvul.002G046800	110bp
PvSTP13.1 RP	CTGAACAGCACCGGAGCATA		
PvSTP13.2 FP	TATCCTGAAGCGCAGGAACC	Phvul.007G055100	114bp
PvSTP13.2 RP	GTGTTGAAAAGAACCGGGGC		

Supplementary Table 3: Differential expression of *PvSTP13.1* and *PvSTP13.2* in the seed coat, embryo and source leaf of *Phaseolus vulgaris*. These data are the mean of four biological replicates with two technical replicates. The data is presented as the mean ± standard error.

**Gene**	**Tissue**	**Mean Ct**	**ΔCt**	**ΔΔCt**	**$2^{-\Delta\Delta Ct}$**
PvSTP13.1	11 DPA Seed coat	22.70 ± 0.11	2.69 ± 0.12	0.00	1.00
	16 DPA Seed coat	21.80 ± 0.05	2.70 ± 0.07	0.004 ± 0.069	1.00 ± 0.04
	21 DPA Seed coat	21.87 ± 0.17	2.36 ± 0.09	-0.34 ± 0.09	1.27 ± 0.08
	26 DPA Seed coat	23.25 ± 0.20	3.21 ± 0.12	0.52 ± 0.12	0.70 ± 0.06
	31 DPA Seed coat	25.81 ± 0.30	5.48 ± 0.23	2.78 ± 0.23	0.15 ± 0.02
	11 DPA Embryo	27.15 ± 0.34	5.92 ± 0.28	3.23 ± 0.28	0.11 ± 0.02
	16 DPA Embryo	30.28 ± 0.46	7.71 ± 0.33	5.02 ± 0.33	0.03 ± 0.01
	21 DPA Embryo	31.30 ± 0.31	10.53 ± 0.08	7.84 ± 0.08	0.0044 ± 0.0002
	26 DPA Embryo	Not detected			
	31 DPA Embryo	Not detected			
	21 DPA Source leaf	21.94 ± 0.14	1.27 ± 0.12	-1.42 ± 0.12	2.71 ± 0.24
PvSTP13.2	11 DPA Seed coat	30.62 ± 0.32	10.55 ± 0.27	7.86 ± 0.27	0.0045 ± 0.0009
	16 DPA Seed coat	29.46 ± 0.16	10.08 ± 0.07	7.39 ± 0.27	0.0060 ± 0.0003
	21 DPA Seed coat	31.19 ± 0.52	11.69 ± 0.32	9.00 ± 0.32	0.0021 ± 0.0004
	26 DPA Seed coat	31.43 ± 0.43	11.29 ± 0.18	8.60 ± 0.18	0.0026 ± 0.0003
	31 DPA Seed coat	32.30 ± 0.54	12.16 ± 0.53	9.47 ± 0.53	0.0016 ± 0.0006
	11 DPA Embryo	30.09 ± 0.30	9.49 ± 0.15	6.80 ± 0.15	0.0091 ± 0.0010
	16 DPA Embryo	29.39 ± 0.25	7.21 ± 0.15	4.51 ± 0.15	0.0445 ± 0.0047
	21 DPA Embryo	28.60 ± 0.32	7.40 ± 0.13	4.70 ± 0.13	0.0388 ± 0.0035
	26 DPA Embryo	30.63 ± 0.39	9.72 ± 0.32	7.03 ± 0.32	0.0081 ± 0.0016
	31 DPA Embryo	Not detected			
	21 DPA Source leaf	27.93 ± 0.26	6.96 ± 0.22	4.26 ± 0.22	0.0527 ± 0.0098

```
                   10        20        30        40        50        60        70        80        90       100
Consensus  MAGGGFAXXA---GGXEFEAKITPIVIISCIMAATGGLMFGYDVGVSGGVTSMXPFLKKFFPVVYRKTVEEKGXDSNYCKYDNQGLQLFTSSLYLAGLTA
AtSTP10    MAGGAFVSEGGG-GGRSYEGGVTAFVIMTCIVAAMGGLLFGYDLGISGGVTSMEEFLTKFFPQVESQMKKAKH-DTAYCKFDNQMLQLFTSSLYLAALVA
AtSTP13    MTGGGFATSA---NGVEFEAKITPIVIISCIMAATGGLMFGYDVGVSGGVTSMPDFLEKFFPVVYRKVVAGADKDSNYCKYDNQGLQLFTSSLYLAGLTA
BvSTP13    MAGGGLAAPVNGAGGVVFEAKITPIVIISCIMAATGGLMFGYDVGISGGVTSMDPFLKKFFPVVFRKKNLPNANQSNYCEYDNEGLQLFTSSLYLAALIA
MdSTP13a   MAGGGFSAGP---GGREFEAKITPIVIISCIMAATGGLMFGYDVGVSGGVTSMSPFLKKFFPVVYRRT-QEQGINSNYCKYDNQGLQLFTSSLYLAGLTA
MtSTP13.1  MAGGGFATS----GGGEFEAKITPIVIISCIMAATGGLMFGYDVGVSGGVTSMHPFLKKFFPAVYRKTVLEAGLDSNYCKYDNQGLQLFTSSLYLAALTS
MtSTP13.2  MTGGGFSGG----NDREFEAKITPIIIISCIMAATGGLMFGYDVGVSGGVASMPPFLKKFFPTVLRQTTESDGSESNYCKYDNQGLQLFTSSLYLAGLTV
PvSTP13.1  MAGGGFTSGA---AGGEFEAKITPIVIISCIMAATGGLMFGYDVGVSGGVTSMAPFLKKFFPTVYRKTVEEKELDSNYCKYDNQGLQLFTSSLYLAGLVA
PvSTP13.2  ---------MTTTSVNDSEAKITPIVILSCIMAATGGLMFGYDVGVSGGVTSMAAFLKEFFPEVYRKTVEEKVVDSNYCKYDNEKLQLFTSCLYLAGLTA
TaSTP13    MPGGGFAVSAP--SGVEFEAKITPIVIISCIMAATGGLMFGYDVGISGGVTSMDDFLREFFPAVLRRKNQDK--ESNYCKYDNQGLQLFTSSLYLAGLTA

                  110       120       130       140       150       160       170       180       190       200
Consensus  TFFASYTTRKLGRRLTMLIAGFFFIXGVALNAAAQNLAMLIXGRILLGCGVGFANQAVPVFLSEIAPXRIRGALNILFQLNVTIGILFANLVNYGTNKIK
AtSTP10    SFMASVITRKHGRKVSMFIGGLAFLIGALFNAFAVNVSMLIIGRLLLGVGVGFANQSTPVYLSEMAPAKIRGALNIGFQMAITIGILVANLINYGTSKMA
AtSTP13    TFFASYTTRTLGRRLTMLIAGVFFIIGVALNAGAQDLAMLIAGRILLGCGVGFANQAVPLFLSEIAPTRIRGGLNILFQLNVTIGILFANLVNYGTAKIK
BvSTP13    TFFASYTTRQLGRRLTMLIAGVFFIVGVALNAAAQDLAMLIVGRLLLGCGVGFANQAVPVFLSEIAPTQIRGGLNILFQLNITIGILFANLVNYGTNKMK
MdSTP13a   TFAASYTTRKFGRKPSMLIAGIFFIVGTILNAAAQDLAMLIIGRISLGCGVGFANQAVPLFLSEIAPTRIRGGLNILFQLNVTLGILFANLVNYGTNKIT
MtSTP13.1  TFFASYTTRTMGRRLTMLIAGFFFIAGVAFNAAAQNLAMLIVGRILLGCGVGFANQAVPVFLSEIAPSRIRGALNILFQLNVTIGILFANLVNYGTNKIS
MtSTP13.2  TFFASYTTRVLGRRLTMLIAGFFFIAGVSLNASAQNLLMLIVGRVLLGCGIGFANQAVPVFLSEIAPSRIRGALNILFQLDITLGILFANLVNYATNKIK
PvSTP13.1  TFFASYTTRRLGRRLTMLIAGFFFLCGVVLNAAAQDLAMLIVGRILLGCGVGFANQAVPVFLSEIAPSRIRGALNILFQLNVTIGILFANLVNYGTNKIK
PvSTP13.2  TFLASHITRKQGRRATMLIAGFIFIAGVAFNAAAQNLAMLIIGRLLLGCGVGFANQAVPVFLSEIAPSRIRGALNILFQLNITLGILFANLVNYATNKIK
TaSTP13    TFFASYTTRRLGRRLTMLIAGVFFIIGVIFNGAAQNLAMLIIGRILLGCGVGFANQAVPLFLSEIAPTRIRGGLNILFQLNVTIGILFANLVNYGTSKIH

                  210       220       230       240       250       260       270       280       290       300
Consensus  GGWGWRLSLGLAGIPALLLTLGALLVVDTPNSLIERGRLEEGKAVLKRIRGTDNIEPEFLELVEASRVAKEVKHPFRNLLKRRNRPQLVISXALQIFQQF
AtSTP10    QH-GWRVSLGLAAVPAVVMVIGSFILPDTPNSMLERGKNEEAKQMLKKIRGADNVDHEFQDLIDAVEAAKKVENPWKNIMESKYRPALIFCSAIPFFQQI
AtSTP13    GGWGWRLSLGLAGIPALLLTVGALLVTETPNSLVERGRLDEGKAVLRRIRGTDNVEPEFADLLEASRLAKEVKHPFRNLLQRRNRPQLVIAVALQIFQQC
BvSTP13    GGNGWRVSLGLAGIPACLLTLGALIVTDTPNSLIERGRLEEGKAVLRRIRGTDNIEPEFLELVEASRMAQQVKHPFRNLLQRRNRPQLVISVAMQVFQQF
MdSTP13a   GGWGWRVSLGLAGFPAGLLTLGALFVVETPNSLVERGLLEQGKSVLKRIRGTENVEPEFLELVEASRIAKEVKHPFRNLLKRRNRPQLIIAVALQIFQQF
MtSTP13.1  GGWGWRLSLGLAGIPALLLTVGAIVVVDTPNSLIERGRLEEGKAVLKKIRGTDNIEPEFLELCEASRVAKEVKHPFRNLLKRKNRPQLIISIALQIFQQF
MtSTP13.2  GHWGWRISLGLGGIPALLLTLGAYLVVDTPNSLIERGHLDKGKAVLRKIRGTDNIEPEFLELVEASRVAKEVKHPFRNLLKRNNRPQLVISIALMIFQQF
PvSTP13.1  GGWGWRLSLGLAGIPAVLLTVGALLVVDTPNSLIERGRLEEGKAVLKKIRGTDNIEPEFLELVEASRVAKEVKHPFRNLLKRRNRPQIVISIALQIFQQF
PvSTP13.2  GGWGWRLSLGLGGLPALLLTLGAFLVVDTPNSLIERGHLEEGKAVLRRIRGIDNIEPEFLELVDACTVAREVKHPFRNILKRRNRPQLLISIALQVFQQF
TaSTP13    P-WGWRLSLSLAGIPAAMLTLGALFVTDTPNSLIERGHLEEGKAVLKRIRGTDNVEPEFNEIVEASRIAQEVKHPFRNLLQRRNRPQLVIAVLLQIFQQF

                  310       320       330       340       350       360       370       380       390       400
Consensus  TGINAIMFYAPVLFNTLGFGNDASLYSAVITGAVNVLSTVVSIYSVDKLGRRLLLLEAGVQMFLSQVVIAIILGIKV-XDHSDDLSKGFAILVVVXVCTF
AtSTP10    TGINVIMFYAPVLFKTLGFGDDAALMSAVITGVVNMLSTFVSIYAVDRYGRRLLFLEGGIQMFICQLLVGSFIGARFGTSGTGTLTPATADWILAFICVY
AtSTP13    TGINAIMFYAPVLFSTLGFGSDASLYSAVVTGAVNVLSTLVSIYSVDKVGRRVLLLEAGVQMFFSQVVIAIILGVKV-TDTSTNLSKGFAILVVVMICTY
BvSTP13    TGINAIMFYAPVLFNTLGFGANASLYSAVITGAVNVLSTVVSIYSVDKLGRRILLLQAGVQMFISQLIIAIILGLKV-KDHSNSLSHGYAIFVVILVCTF
MdSTP13a   TGINAIMFYAPVLFNTLGFGNDAALYSAVITGAVNVLSTVVSIYSVDKVGRRLLLLQAGVQMFISQVAIAIILGIKV-KDHSDDLHKGFAIFVVVLICTY
MtSTP13.1  TGINAIMFYAPVLFNTVGFKNDASLYSAVITGAVNVLSTIVSIYFVDKLGRRMLLLEAGVQMFLSQIVIAIILGIKV-TDHSDDLSKGYAIFVVILVCTF
MtSTP13.2  TGINAIMFYAPVLFNTLGFKNDAALYSAVITGAINVISTIVSIYSVDKLGRRKLLLEAGVQMLLSQMVIAIVLGIKV-KDHSEELSKGYAALVVVMVCIF
PvSTP13.1  TGINAIMFYAPVLFSTVGFGNDAALYSAVITGAVNVLSTVVSIYSVDKLGRRLLLLEAGVQMFLSQVVIAIILGIKV-KDDSNDLSKGFAVLVVVLVCTF
PvSTP13.2  TGINAIMFYAPVLFNTLGFKNDASLYSAVITGAVNVISTVVSIYSVDRLGRRMLLLEAGVQMFLSQFVIAVIIGMKV-RDHDEDLSKGFAVLVVVMVCIF
TaSTP13    TGINAIMFYAPVLFNTLGFKSDASLYSAVITGAVNVLATLVSVYAVDRAGRRALLLEAGVQMFLSQVVIAVVLGIKV-TDKSDNLGHGWAILVVVMVCTY

                  410       420       430       440       450       460       470       480       490       500
Consensus  VSAFAWSWGPLGWLIPSETFPLETRSAGQSVTVCVNLLFTFVIAQAFLSMLCHFKFGIFLFFSGWVLIMSVFVLFLLPETKNVPIEEMTERVWKQHWFWK
AtSTP10    VAGFAWSWGPLGWLVPSEICPLEIRPAGQAINVSVNMFFTFLIGQFFLTMLCHMKFGLFYFFASMVAIMTVFIYFLLPETKGVPIEEM-GRVWKQHWFWK
AtSTP13    VAAFAWSWGPLGWLIPSETFPLETRSAGQSVTVCVNLLFTFIIAQAFLSMLCHFKFGIFIFFSAWVLIMSVFVMFLLPETKNIPIEEMTERVWKKHWFWA
BvSTP13    VSSFAWSWGPLGWLIPSEIFPLETRSAGQSVTVCVNMLFTFIIGQAFLSMLCKFKFGIFLFFSGWVLIMSIFVVFLIPETKNVPIEEMTERVWKNHWLWK
MdSTP13a   VAAFAWSWGPLGWLIPSETFPLETRSAGQSLTVCTNLLFTFVIAQGFLSMLCHFKYGIFLFFSGWVLVMSFFVLFLLPETKNIPIEEMTERVWKQHWLWK
MtSTP13.1  VSAFAWSWGPLGWLIPSETFPLETRSAGQSVTVCVNMLFTFVIAQAFLSMLCHFKFGIFLFFSGWVLIMSIFVLFLVPETKNIPIEEMTERVWKQHWFWK
MtSTP13.2  VSAFAWSWGPLAWLIPSEIFPLETRSAGQSVTVCVNFLFTAVIAQAFLSMLCYFKFGIFFFFSGWILFMSTFVFFLVPETKNVPIEEMTQRVWKQHWFWK
PvSTP13.1  VSSFAWSWGPLGWLIPSETFPLETRSAGQSVTVCVNLLFTFVIAQAFLSMLCHFKFGIFLFFSGWVFIMSFFVLFLLPETKNVPIEEMTERVWKQHWFWK
PvSTP13.2  VSAFAWSWGPLSWLIPSEIFPLETRSAGQSIAVCVNLLCTFFIAQAFLSMLCIFKFGIFLFFSGCVLIMSTFVLLLLPETKNVPIEEMTERVWKQHWLWK
TaSTP13    VASFAWSWGPLGWLIPSETFPLETRSAGQSVTVCVNLLFTFLIAQAFLSMLCHLKFAIFIFFSAWVLVMSVFVLFFLPETKNVPIEEMTDKVWKQHWFWK

                  510       520       530
Consensus  RFMXDDXIHXEKVDGXXNKXNXXSNLXXGXXXXSAAAL
AtSTP10    KYIPEDAIIGGHDDNNTN--------------------
AtSTP13    RFMDDHNDHEFVNGEKSNGKSNGFDPSTRL--------
BvSTP13    RYMEDDHRYRDGIEVNAIKNAQKNNKVGGQNNGSAAAL
MdSTP13a   RFMDDDDYHIEGNHGDGLKKNGQANGFDGVSQL-----
MtSTP13.1  RFMEDDNEKVSNADYPKIKNNPNSQL------------
MtSTP13.2  RFVENDYIEDEKVTGGNSPSRNDLVSQL----------
PvSTP13.1  RFIDDDYTADEKVANVSNGYDPASRL------------
PvSTP13.2  RFAEDDCVKEEKVVTDI---------------------
TaSTP13    RFMDDDDHHHNIANGKNATV------------------
```

Supplementary Figure 1: Amino acid sequence alignment of AtSTP10 (At3g19940), AtSTP13 (At5G26340) BvSTP13 (Bevul.3G139500), MdSTP13a (MD13G1189100), MtSTP13.1 (Medtr5g006070), MtSTP13.2 (Medtr1g104780), PvSTP13.1 (Phvul.002G046800), PvSTP13.2 (Phvul.007G055100) and TaSTP13 (Traes_4DL_CFC191A06). Amino acid residues encased in a red box indicate residues participating in AtSTP10 binding of sugar (Phe39, Leu43, Gln177, Ile184, Gln295, Gln296, Asn301, Asn332, Phe401, Gly406, Trp410, Asn433, Thr437; Paulsen et al. 2019). Amino acid residues encased in a blue box indicate the proton donor-acceptor pair (Asp42 and Arg142) essential for AtSTP10 transport capability (Paulsen et al. 2019). Amino acid residues encased in a green box indicate the tri-aromatic motif required for endoplasmic reticulum export and plasma membrane localisation of STPs (Yamada et al. 2017).
