# Supplementary material for: Phaseolus vulgaris STP13.1 is an H+‐coupled monosaccharide transporter, present in source leaves and seed coats, with higher substrate affinity at depolarized potentials
Source: Plant Direct. 2024 Apr 22;8(4):e585. doi: 10.1002/pld3.585 (PMC11033725; doi:10.1002/pld3.585)
Supplement: Supplementary file 1 — Data S1 Supporting Information [file PLD3-8-e585-s002.pdf]

Plant Direct
Open Access

Phaseolus vulgaris STP13.1 is a H⁺-coupled monosaccharide transporter, present in source leaves and seed coats, with higher substrate affinity at depolarised potentials.

Functional characterisation of PvSTP13.1

Joseph L. Pegler[1,*], John W. Patrick[1], Benjamin McDermott[1], Anthony Brown[1], Jackson MJ. Oultram[1], Christopher PL. Grof[1,+] and John M. Ward[2,+]

Decision Letter Round 1:

October 29, 2023

Dr. Joseph Pegler
University of Newcastle
Callaghan, N/A
Australia

RE: Phaseolus vulgaris STP13.1 is a H+-coupled monosaccharide transporter, expressed in seed coats, with higher substrate affinity at depolarised potentials.

Dear Dr. Pegler:

Thank you for submitting to Plant Direct. All required reviews have been returned and we have now finished our evaluation of your manuscript. In light of the reviewers' and editor's comments, further (minor) revisions are needed before the paper can be accepted for publication in Plant Direct.

Please view the editors' and reviewers' comments below and use their suggestions as a guide while you work on your revision.

When uploading the revised version of this article, please be sure to include the following:

-A word document that contains your response to the reviewers. You should respond to each reviewer comment and note the changes made to the manuscript. If you do not agree with a reviewer's comment and choose not to make a suggested revision, please explain why. Please try to provide as complete an answer as possible to each reviewer's criticisms.
-A tracked changes document with each change highlighted
- A clean version of the latest version of the manuscript

To upload your revision, please click the link below.
https://plantdirect.msubmit.net/cgi-bin/main.plex?el=A2Lr2dK2A6oRX3I2A9ftdN2arQEu4cgcM2EH7rBNgwZ

In order to provide as timely a service as possible, we ask that your revision is resubmitted within three months after receipt of this request. If an extension is needed, please send a request, along with a brief explanation, to the editorial office at plantdirect@wiley.com .

Please note that, in addition to publishing reviewer comments, the author's responses to review comments will also be published alongside the final version of the paper. If you would not like the author's responses to be published, please contact the editorial office at plantdirect@wiley.com .

Thank you very much for giving us an opportunity to review your work. I look forward to receiving the next version.

Sincerely,

Hernani Gerós

Hsou-min Li

Editor, Plant Direct

Dear authors, you can benefit from the professional revisions made by both referees, as their comments and suggestions can enhance your manuscript impact. I also concur with both reviewers, who commend your paper's readability and technical soundness. In our lab, we typically assess sugar transport by quantifying the uptake of radioactive sugars in heterologous systems, a method I consider more suitable due to its direct measurement of sugar uptake within the intracellular space. Nevertheless, your indirect measurement has provided valuable insights into transporter kinetics and energetics. Both reviewers concur on the need for an additional experiment to evaluate the expression of PvSTP13.1 and PvSTP13.2 in other plant tissues beyond seed, and I support this recommendation.

Hernâni Gerós

Reviewer comments:
Reviewer #1:

The manuscript is well written, and the authors address fundamental questions in the field of plant sugar transport with original results on the characterization of the transport kinetics of a key transporter candidate PvSTP13.1 from a legume species of agricultural interest (Phaseolus vulgaris) with a proposed role in hexose retrieval back towards the seed coat.

I suggest that the manuscript may be suitable for publication in Plant Direct, given that the authors positively answer the following reviews :

Main critic :
-My main critics would be that the paper sometimes feels unbalanced because of the limited biological evidence based on expression studies, concluding perhaps too prematurely on a putative role for STP13.1 in seed nutrition.

Figures :
-Please provide a vectorized image of figure 1. The current picture is pixelized and it is hard to read/ review this figure, notably the gene names/accessions. Here, it would also be valuable to add a supplemental table with all gene names/accessions and protein sequences.

-Please modify Figure 2. Draw a graph with actual expression values (not using a fold change unit) to better compare expression values between different seed tissues and developmental stages. For instance, I find it unusual that your first expression value in seed coat is used as a reference value (fold change of 1.0) to compare every other values of STP13 expression in seed coat or embryo at other developmental stages.

-It would also be interesting to see the expression pattern of PvSTP13.1 and PvSTP13.2 in other plant tissues (beyond seed) to compare the expression value at the whole plant level.

-Regarding the four individual figures on transport kinetics (Figures 3-6) - Here, I suggest rearranging the figures, maybe moving some info to the supplemental materials or combining parallel information could improve the readability of the manuscript.

Introduction :
-In the Introduction, the authors state "Collectively, these findings suggest that there are two isoforms of STP13; a foliar expressed hexose specific isoform that confers resistance to bacterial and fungal pathogens and a sink expressed isoform that transports both hexoses and sucrose." Here I do not understand how the authors come to this conclusion. For instance, in Arabidopsis, only a single AtSTP13 gene (isoform?) is present, and it is mostly expressed in source leaf.

-Please rewrite the last paragraph of the introduction, opening to your reader what your main points will be and what perspective you will take. As it states, it is rather written as a concluding paragraph.

Material & Methods :
-In the Material & Methods, the authors mention that sequences were retrieved from Malus domestica v1.1 (Daccord et al. 2017). Here, I did not see Malus domestica STP protein on the phylogenetic analysis (fig 1).

-The authors detail the time (in DPA) of pre-storage phase are storage phase, it would also be interesting to have the interval of desiccation phase. Was this period considered in the study ?

-Please add a citation for the reference genes (ALKBH6, HBP, SDHAF1), if these genes were previously

used in a study.

Results & Discussion:
-It would be interesting to add some more info about the hypothetical role or expression profile of the second isoform PvSTP13.2. It is also not very clear why the functional characterization of PvSTP13.2 was not further investigated.

-In the discussion, the authors discuss "the amino acids present in the substrate binding site" and the substrate specificity observed between MdSTP13a, PvSTP13, AtSTP10 and BvSTP13 (Sup Figure2). Here, it would have been interesting to aligned further STP13 sequences, I am thinking for instance of AtSTP13 in Arabidopsis, MtSTP13.1 and .2 in Medicago truncatula , BvSTP13 of Beta vulgaris and TaSTP13 of wheat and other STP13 orthologs.

-The authors conclude this manuscript with these sentences: "Additionally, as STP13 expression has been shown to be responsive to abiotic stress and ABA, it may serve as a signal for source to sink transport of sugars during unfavourable growth conditions (Nørholm et al. 2006; Yamada et al. 2011; Gupta et al. 2021; Reyer et al. 2021; Urwat et al. 2021). With a mounting number of studies indicating STP13 is central to the plant pathogenic response, PvSTP13.1 is positioned as a potential candidate for gene editing of common bean plants for an improved tolerance to species-specific pathogens. "With a mounting number of studies indicating STP13 is central to the plant pathogenic response, PvSTP13.1 is positioned as a potential candidate for gene editing of common bean plants for an improved tolerance to species-specific pathogens." Here I would remove both sentences which are not consistent with the rest of the paragraph and the overall study presented here. The concluding paragraph would be more concise and focused without these sentences.

References:
I found at several instances that some references on STP were missing. Please at least cite and discuss the following references:
-Buttner 2010 The Arabidopsis sugar transporter (AtSTP) family: an update; Plant Biology doi:10.1111/j.1438-8677.2010.00383.x
- Lemonnier et al 2014 Expression of Arabidopsis sugar transport protein STP13 differentially affects glucose transport activity and basal resistance to Botrytis cinerea; Planta DOI 10.1007/s11103-014-0198-5

Reviewer #2:

Comments
This well written and articulated study details the phylogenetic analysis, expression profiling and functional characterization of PvSTP13.1 using the Xenopus oocyte system, where TEVC was used to analyze substrate affinity and substrate selectivity for a host of sugars. 28 PvSTP family members were identified that fell into 8 phylogenetic clades. PvSTP13.1 was expressed in seed coats at all time points

examined and in embryos only 11 and 16 days after anthesis. It is clear from the functional data that PvSTP13.1 has a role in hexose retrieval from the apoplast back to seed coats during seed filling, as appropriate pH and membrane potentials of the target tissues were examined. Functional analysis is very thorough and well described.

Given that some other STP13s are predominantly expressed in photosynthetic tissues, it would greatly add to the story to include a comparison of PvSTP13.1 expression in mature leaf tissue. Especially given talk around source and sink isoforms of STP13. It is very difficult to make any comparisons to the level of PvSTP13.1 leaf expression reported by Urwat et al 2021.

Please clarify whether expression of other STPs was tested in seed coats/embryos and PvSTP13.1 was highest, or was only PvSTP13.1 tested? If no other expression analysis was done, why was PvSTP13.1 chosen over PvSTP13.2?

Of the suite of sugars tested in oocytes, is there any evidence as to which are the most physiologically relevant or most abundant? Or is this more about understanding properties of the transporter itself?

Given the presence of foliar and sink expressed STP13 isoforms are mentioned in the introduction, it would be good to come back to this in the discussion and mention how PvSTP13.1 then fits with the proposition of two isoforms.

Minor comments
Abstract
Could say "transport" or "molecular" function in "To study the function of PvSTP13.1" to differentiate from "physiological" function

Intro
The sentence "In contrast, AtSTP13 and MtSTP13.1" could be changed to "In contrast, AtSTP13 and MtSTP13.1 which are functional transporters, ..." to avoid any confusion here

Confers resistance is a strong terminology here, consider using "contributes to basal defense against microbial pathogens by competing for sugar uptake". Increased resistance was only observed in STP13 overexpressing plants.

Methods
Would be good to mention in the methods how many ng of cRNA was injected so the reader doesn't have to go to Sivitz et al 2005 to find this.
Can you please elaborate on the quantification method used to normalize to 3 genes? Were fold changes determined separately then averaged?

Results
Check first heading, appears to be truncated "The sugar transport protein (STP) family in"

First paragraph, typo "PvSTP13,1" instead of PvSTP13.1

Discussion
Check italicisation of "At" in "AtSTP13" in "determined for AtSTP13 (74 µM at pH 5.0; Nørholm et al. 2006), BvSTP13 (75 µM at pH 5.5; Reyer et"
Might be good to make the final sentence a bit more specific - "PvSTP13.1 is positioned as a potential candidate for gene editing of common bean plants for an improved tolerance to species-specific pathogens." Is this referring to increasing expression or introducing a G144R mutation? Since G144R abolishing the sugar transport function would go against sugar transport role proposed in this study.

Figures
Phylo tree in Figure 1 is not legible at printed size (100% zoom), please ensure high resolution image is provided (apologies if this was already done) and increase font size if possible. Same goes for the alignment text in Supp Figure 1.

Decision Letter Round 2:

March 25, 2024

Dr. Joseph Pegler
University of Newcastle
Callaghan, N/A
Australia

RE: Phaseolus vulgaris STP13.1 is a H+-coupled monosaccharide transporter, expressed in source leaves and seed coats, with higher substrate affinity at depolarised potentials.

Dear Dr. Pegler:

Thank you for submitting to Plant Direct. All required reviews have been returned and we have now finished our evaluation of your manuscript.

Both reviewers and I agreed that your manuscript only needs minor revisions before being acceptable for publication. Please kindly address in the discussion the comment of Reviewer #1 regarding the need for further evidence to support the conclusions drawn regarding STP13.1's role in seed nutrition. You may acknowledge that the findings encourage future studies to explore this intriguing avenue of research, which could provide further insights into the involvement of STP13.1 in seed nutrition.

Please view the remaining reviewers' comments below and use their suggestions as a guide while you work on your revision.

When uploading the revised version of this article, please be sure to include the following:

-A word document that contains your response to the reviewers. You should respond to each reviewer comment and note the changes made to the manuscript. If you do not agree with a reviewer's comment and choose not to make a suggested revision, please explain why. Please try to provide as complete an answer as possible to each reviewer's criticisms.
-A tracked changes document with each change highlighted
- A clean version of the latest version of the manuscript

To upload your revision, please click the link below.
https://plantdirect.msubmit.net/cgi-bin/main.plex?el=A5Lr1dK2B3oRX2I6A9ftdN2arQEu4cgcM2EH7rBNgwZ

In order to provide as timely a service as possible, we ask that your revision is resubmitted within three months after receipt of this request. If an extension is needed, please send a request, along with a brief explanation, to the editorial office at plantdirect@wiley.com .

Please note that, in addition to publishing reviewer comments, the author's responses to review comments will also be published alongside the final version of the paper. If you would not like the author's responses to be published, please contact the editorial office at plantdirect@wiley.com .

Thank you for giving us the opportunity to review your work. I look forward to receiving the next version with only minor modifications.

Sincerely,

Hernani Gerós

Hsou-min Li

Editor, Plant Direct

Reviewer #1:

An answer letter to the reviewer comments is missing. However, the author seems to have addressed most comments in the revised version of the manuscript.

-My main critic remains unanswered: "the paper sometimes feels unbalanced because of the limited biological evidence based on expression studies, concluding perhaps too prematurely on a putative role for STP13.1 in seed nutrition."

-The third internal reference gene (initially SDHAF1) was modified to IDE in the revised version without any explanation/answer given to the editor/reviewers.

-The following reference is still missing: Buttner 2010 The Arabidopsis sugar transporter (AtSTP) family: an update; Plant Biology doi:10.1111/j.1438-8677.2010.00383.x

Reviewer #2:

I thank the authors for considering my comments. I am satisfied that points raised in my review have been adequately addressed, particularly on the expression of PvSTP13.1 in source tissues and justification of selection of this gene for detailed functional characterization. I have some very minor considerations below to address:

The word "expressed" in the title denotes a gene which should be italicized. Otherwise, consider "present" or another descriptor to refer to the protein

Methods - Plant Growth conditions
Course river sand should be "Coarse" river sand
Please check the ordering of units in the following sentence as they are inconsistent: During light hours, plants were chronologically illuminated with 3h/200µmol s^{-1} m^{-2} s^{-1} photosynthetic active radiation (PAR), 8h/400µmol s^{-1} m^{-2} PAR and 3h/200µmol s^{-1} m^{-2} PAR.
Please check italicisation of "MluI" in oocyte methods

Discussion
Please consider revising "localisation to the source leaves" in the final paragraph as expression was measured rather than localization performed
Please remove hyphen after leaf in "hexoses from the leaf-" (unless there was meant to be one in seed coat-apoplasm)

Figures
Figure 6: Typo on panel labelling - "substarte" is used instead of "substrate" above panel D

Decision Letter Round 3:

March 28, 2024
Dr. Joseph Pegler
University of Newcastle
Callaghan, N/A
Australia

MSID: 2023-01306R2
MS TITLE: Phaseolus vulgaris STP13.1 is a H+-coupled monosaccharide transporter, present in seed coats, with higher substrate affinity at depolarised potentials.

Plant Direct
Open Access

Dear Dr. Joseph Pegler:

I am pleased to inform you that your manuscript "Phaseolus vulgaris STP13.1 is a H+-coupled monosaccharide transporter, present in seed coats, with higher substrate affinity at depolarised potentials." has been accepted for publication in Plant Direct.

Your article will appear online in the next available issue of Plant Direct. To ensure your article gets published as quickly as possible, please pay attention to the steps detailed below. We have found that most of the delays happen at this stage, especially at the payment stage, so please respond as quickly as possible when prompted.

License Agreement: Once your article has been accepted it will move to Production and undergo admin and file checking - you may receive an email with any queries we have at this stage. When all required items are received by the publisher and queries resolved, the corresponding author will receive an email from Wiley's Author Services system which will ask them to log on at https://authorservices.wiley.com/bauthor and will present them with the appropriate license for completion. Your article cannot be published until both the signed license agreement and payment of the article fee have been received.

Payment of the Open Access Article Publication Fee: All articles published in Plant Direct are fully open access: immediately and freely available to read, download and share. Plant Direct charges a publication fee to cover the publication costs. The corresponding author for this manuscript should have already received a quote from the payments team (cs-openaccess@wiley.com) with the estimated article publication fee; please email cs-openaccess@wiley.com if this has not been received. The corresponding author should log on to the Wiley Author Services site, where the publication fee can be paid by credit card or an invoice. Pro Forma can also be requested. Payment of the publication charge must be received before the article will be published online.

Proofs: You will have the opportunity to look over your paper once more when you receive the author proofs for your article. The proofs will be with you in approximately two weeks. Please note that, in addition to publishing reviewer comments, the author's responses to review comments will also be published alongside the final version of the paper. If you would not like the author's responses to be published, please contact the editorial office at plantdirect@wiley.com .

Promotion of your article: You can help your research get the attention it deserves! Check out Wiley's free Promotion Guide for best-practice recommendations for promoting your work at www.wileyauthors.com/eeo/guide. And learn more about Wiley Editing Services which offers professional video, design, and writing services to create shareable video abstracts, infographics, conference posters, lay summaries, and research news stories for your research at www.wileyauthors.com/eeo/promotion.

Thank you again for your contribution to Plant Direct. If you have any questions, feel free to contact the editorial office at plantdirect@wiley.com .

Sincerely,

Hernani Gerós

Hsou-min Li

Editor, Plant Direct

Author’s response to First Decision:

Dear Professors Hernani Gerós and Hsou-min Li, Thank you for considering our manuscript "Phaseolus vulgaris STP13.1 is a H+-coupled monosaccharide transporter, expressed in seed coats, with higher substrate affinity at depolarised potentials" for publication in Plant Direct. We would like to thank you and the reviewers for providing constructive comments that helped us improve the manuscript. We have addressed each comment from Reviewer 1 and 2, and provided a response indicating changes that were made in the revised manuscript. Reviewer #1: 1. My main critics would be that the paper sometimes feels unbalanced because of the limited biological evidence based on expression studies, concluding perhaps too prematurely on a putative role for STP13.1 in seed nutrition. Response: We appreciate and agree with the point made by Reviewer 1. However, as acknowledged in the Introductory text (see Para 3), progress in understanding the role of STPs in developing seeds has been thwarted by their functional redundancy conferred by co-expression of many STP sub-clades. Therefore, like other papers on STPs in developing seeds (see Pegler et al 2023), we have relied on relative expression levels of STP13.1 to speculate about its possible role in seed filling. To this end, where appropriate, we have modified the text to indicate its speculative nature. 2. Please provide a vectorized image of Figure 1. The current picture is pixelized and it is hard to read/ review this figure, notably the gene names/accessions. Here, it would also be valuable to add a supplemental table with all gene names/accessions and protein sequences. Response: We apologise for this oversight. To rectify this issue, Figure 1 has been reconstructed containing only the gene names as a high quality TIF file. The gene ID associated with each gene has been provided in Supplementary Table 1 which is now referred to in the Figure 1 legend. 3. Please modify Figure 2. Draw a graph with actual expression values (not using a fold change unit) to better compare expression values between different seed tissues and developmental stages. For instance, I find it unusual that your first expression value in seed coat is used as a reference value (fold change of 1.0) to compare every other values of STP13 expression in seed coat or embryo at other developmental stages. Response: Relative fold change using the 2-ΔΔCt is a commonly used approach for comparing expression values between spatially and/ or temporally distinct samples. The data presented in Figure 2 confirms and readily displays to the reader PvSTP13.1 is predominately expressed in the seed coat and subtending source leaf in comparison to the embryo. For this reason, relative fold change has been retained for Figure 2. The addition of Supplementary Table 3 provides the raw Ct values, ΔCt and ΔΔCt for STP13.1 and STP13.2 in all assessed samples. 4. It would also be interesting to see the expression pattern of PvSTP13.1 and PvSTP13.2 in other plant tissues (beyond seed) to compare the expression value at the whole plant level. Response: We agree. As the

objective of this study is framed to functionally characterise the transport mechanism of STP13.1 expressed in seeds and infer a possible role in seed loading it is logical to include testing for its expression in source leaves. To this end expression of PvSTP13.1 has been additionally assessed in the subtending source leaf of the targeted pods and this data is presented in Figure 2. The expression of PvSTP13.2 in seed coat, embryo and source leaf is provided in Supplementary Table 3. 5. Regarding the four individual figures on transport kinetics (Figures 3-6) - Here, I suggest rearranging the figures, maybe moving some info to the supplemental materials or combining parallel information could improve the readability of the manuscript. Response: As the transport kinetics (Figure 3-6) is central to this manuscript we respectfully believe the data is best left in the body of the manuscript to reduce the necessity for the reader to access Supplementary materials. 6. In the Introduction, the authors state "Collectively, these findings suggest that there are two isoforms of STP13; a foliar expressed hexose specific isoform that confers resistance to bacterial and fungal pathogens and a sink expressed isoform that transports both hexoses and sucrose." Here I do not understand how the authors come to this conclusion. For instance, in Arabidopsis, only a single AtSTP13 gene (isoform?) is present, and it is mostly expressed in source leaf. Response: We appreciate and agree with Reviewer 1 that this statement is an overreach. This sentence has been removed from the manuscript. 7. Please rewrite the last paragraph of the introduction, opening to your reader what your main points will be and what perspective you will take. As it states, it is rather written as a concluding paragraph. Response: We agree with and thank Reviewer 1 for raising this issue. The last paragraph of the introduction has been rewritten to address this issue. 8. In the Material & Methods, the authors mention that sequences were retrieved from Malus domestica v1.1 (Daccord et al. 2017). Here, I did not see Malus domestica STP protein on the phylogenetic analysis (fig 1). Response: MdSTP13a was not included in Fig. 1 however, it was included in the amino acid sequence alignment presented in Supplementary figure 1. 9. The authors detail the time (in DPA) of pre-storage phase are storage phase, it would also be interesting to have the interval of desiccation phase. Was this period considered in the study? Response: The inclusion of a seed desiccation interval was not included as part of this study. Our primary interest is in seed filling that ceases once development enters the desiccation phase. The selection of these specific time points across the prestorage and storage phases of seed development were selected based on the previous identification of these phases in our selected cultivar by Thomas et al., (2000). 10. Please add a citation for the reference genes (ALKBH6, HBP, SDHAF1), if these genes were previously used in a study. Response: These reference genes have not been used in a previous published study. These genes have been selected from 20 assessed candidates identified to have the highest stability across the embryo, seed coat and source leaf of Phaseolus vulgaris. A manuscript is currently under preparation pertaining to the identification and characterisation of these housekeeping genes. IDE has been used in place of SDHAF1 due to its higher stability in the newly included source leaf tissue. 11. It would be interesting to add some more info about the hypothetical role or expression profile of the second isoform PvSTP13.2. It is also not very clear why the functional characterization of PvSTP13.2 was not further investigated. Response: The expression profile of PvSTP13.2 is now provided in Supplementary Table 3. It was not further investigated and its hypothetical role was not discussed in this manuscript because of its very low expression levels comparable to PvSTP13.1 across each of the assessed samples. 12. In the discussion, the authors discuss "the amino acids present in the substrate binding site" and the substrate specificity observed between MdSTP13a, PvSTP13, AtSTP10 and BvSTP13 (Sup Figure2). Here, it would have been interesting to aligned further STP13 sequences, I am thinking for instance of AtSTP13 in

Plant Direct

Open Access

Arabidopsis, MtSTP13.1 and .2 in Medicago truncatula, BvSTP13 of Beta vulgaris and TaSTP13 of wheat and other STP13 orthologs. Response: AtSTP13, MtSTP13.1, MtSTP13.2 and TaSTP13 have been included into the amino acid alignment presented in Supplementary Figure 1. 13. The authors conclude this manuscript with these sentences: "Additionally, as STP13 expression has been shown to be responsive to abiotic stress and ABA, it may serve as a signal for source to sink transport of sugars during unfavourable growth conditions (Nørholm et al. 2006; Yamada et al. 2011; Gupta et al. 2021; Reyer et al. 2021; Urwat et al. 2021). With a mounting number of studies indicating STP13 is central to the plant pathogenic response, PvSTP13.1 is positioned as a potential candidate for gene editing of common bean plants for an improved tolerance to species-specific pathogens. Here I would remove both sentences which are not consistent with the rest of the paragraph and the overall study presented here. The concluding paragraph would be more concise and focused without these sentences. Response: We thank Reviewer 1 for their comment and agree. These sentences have been removed. 14. I found at several instances that some references on STP were missing. Please at least cite and discuss the following references: -Buttner (2010) The Arabidopsis sugar transporter (AtSTP) family: an update; Plant Biology doi:10.1111/j.1438-8677.2010.00383.x - Lemonnier et al., (2014) Expression of Arabidopsis sugar transport protein STP13 differentially affects glucose transport activity and basal resistance to Botrytis cinerea; Planta DOI 10.1007/s11103-014-0198-5 Response: The role of AtSTP13 in modulating plant susceptibility to pathogen infection has been investigated by Lemonnier et al., (2014) and Yamada et al., (2016), for this reason, these references have been added to the introduction. While Buttner (2010) elegantly summarised what was known about the Arabidopsis STP family at that point in time, we believe the references we have selected with respect to AtSTP10 and AtSTP13 are most appropriate. Reviewer #2: 15. Given that some other STP13s are predominantly expressed in photosynthetic tissues, it would greatly add to the story to include a comparison of PvSTP13.1 expression in mature leaf tissue. Especially given talk around source and sink isoforms of STP13. It is very difficult to make any comparisons to the level of PvSTP13.1 leaf expression reported by Urwat et al 2021. Response: We agree with the point made by Reviewer 2. To this end expression of PvSTP13.1 has been additionally assessed in source leaves subtending pods at 21 DPA and this data is presented in Figure 2. The addition of Supplementary Table 3 provides the raw Ct values for PvSTP13.1 and PvSTP13.2 showing comparable PvSTP13.1 expression levels to those reported by Urwat et al 2021. 16. Please clarify whether expression of other STPs was tested in seed coats/embryos and PvSTP13.1 was highest, or was only PvSTP13.1 tested? If no other expression analysis was done, why was PvSTP13.1 chosen over PvSTP13.2? Response: We have now amended the last paragraph in the Introduction that includes the rationale for focusing on STP13s. The expression profile of PvSTP13.2 is now provided in Supplementary Table 3. It was not further investigated because of its very low expression level compared to PvSTP13.1 in all of the assessed samples of this study. 17. Of the suite of sugars tested in oocytes, is there any evidence as to which are the most physiologically relevant or most abundant? Or is this more about understanding properties of the transporter itself? Response: During the pre-storage phase of seed development, the apoplasmic space bathing the developing embryo is dominated by the hexoses, glucose and fructose (up to 100 mM). This arises due to high cell wall invertase activity necessary to maintain sink strength during early stages of embryo development. The large suite of sugars tested (Fig. 4-6) was to ensure PvSTP13.1 was functionally characterised thoroughly. 18. Given the presence of foliar and sink expressed STP13 isoforms are mentioned in the introduction, it would be good to come back to this in the discussion and mention how PvSTP13.1 then fits with the proposition of two isoforms. Response: This sentence has

been removed from the manuscript as we agree with Reviewer 1, it was an overreach. 19. Abstract: Could say "transport" or "molecular" function in "To study the function of PvSTP13.1" to differentiate from "physiological" function Response: We thank the reviewer for their suggestion to improve the specificity of this sentence and have added ‘molecular’. 20. Intro: The sentence "In contrast, AtSTP13 and MtSTP13.1" could be changed to "In contrast, AtSTP13 and MtSTP13.1 which are functional transporters, ..." to avoid any confusion here Response: We thank the reviewer for identifying the ambiguity of this sentence and have added revised the sentence 21. Intro: Confers resistance is a strong terminology here, consider using "contributes to basal defense against microbial pathogens by competing for sugar uptake". Increased resistance was only observed in STP13 overexpressing plants. Response: We thank the reviewer for their suggestion which is incorporated into the text. 22. Would be good to mention in the methods how many ng of cRNA was injected so the reader doesn't have to go to Sivitz et al 2005 to find this. Response: The text now indicates 50 nl (1.1 ng nL^{-1}) of cRNA was injected into each oocyte. 23. Can you please elaborate on the quantification method used to normalize to 3 genes? Were fold changes determined separately then averaged? Response: The average Ct value of the three housekeeping genes was used to determine the ΔCt of each biological replicate. The fold change of each sample was then determined relative to the average ΔCt of the 11DPA seed coat sample using the 2-ΔΔCt. 24. Results: Check first heading, appears to be truncated "The sugar transport protein (STP) family in" First paragraph, typo "PvSTP13,1" instead of PvSTP13.1 Response: The title reads “The sugar transport protein (STP) family in Phaseolus vulgaris”. Thank you for identifying this typo, it has now been rectified. 25. Discussion: Check italicisation of "At" in "AtSTP13" in "determined for AtSTP13 (74 µM at pH 5.0; Nørholm et al. 2006), BvSTP13 (75 µM at pH 5.5; Reyer et" Might be good to make the final sentence a bit more specific - "PvSTP13.1 is positioned as a potential candidate for gene editing of common bean plants for an improved tolerance to species-specific pathogens." Is this referring to increasing expression or introducing a G144R mutation? Since G144R abolishing the sugar transport function would go against sugar transport role proposed in this study. Response: Thank you for identifying this oversight. “At” is no longer italicised. In agreeance with a point raised by Reviewer 1, this sentence has been removed from the manuscript to make the concluding paragraph more concise. 26. Figures: Phylo tree in Figure 1 is not legible at printed size (100% zoom), please ensure high resolution image is provided (apologies if this was already done) and increase font size if possible. Same goes for the alignment text in Supp Figure 1. Response: We apologise for this oversight. To rectify this issue, Figure 1 has been reconstructed containing only the gene names as a high quality TIF file. The gene ID associated with each gene has been provided in a Supplementary Table 1 which is now indicated in the Figure 1 legend. Supplementary Figure 1 is now presented in a portrait orientation to maximise the size. The resolution of this image has also been increased.

Author’s response to Second Decision:

Dear Professors Hernani Gerós and Hsou-min Li, Thank you for considering our manuscript "Phaseolus vulgaris STP13.1 is a H+-coupled monosaccharide transporter, expressed in source leaves and seed coats, with higher substrate affinity at depolarised potentials" for publication in Plant Direct. We would like to thank you and the reviewers for providing constructive comments that helped us improve the manuscript. We have addressed each comment from Reviewer 1 and 2, and provided a response

indicating changes that were made in the revised manuscript. Reviewer #1: 1. An answer letter to the reviewer comments is missing. However, the author seems to have addressed most comments in the revised version of the manuscript. My main critic remains unanswered: "the paper sometimes feels unbalanced because of the limited biological evidence based on expression studies, concluding perhaps too prematurely on a putative role for STP13.1 in seed nutrition." Response: Unfortunately, we are unsure why Reviewer 1 has not received a copy of our response to reviewer and editor comments. Please note a document containing detailed descriptions of manuscript revisions or responses to each reviewer comment was uploaded alongside the first revision of the manuscript. We appreciate and agree with the point made by Reviewer 1. However, as acknowledged in the Introductory text (see Para 3), progress in understanding the role of STPs in developing seeds has been thwarted by their functional redundancy conferred by co-expression of many STP subclades. Therefore, like other papers on STPs in developing seeds (see Pegler et al 2023), we have relied on relative expression levels of STP13.1 to speculate about its possible role in seed filling. To this end, where appropriate, we have modified the text to indicate its speculative nature. 2. The third internal reference gene (initially SDHAF1) was modified to IDE in the revised version without any explanation/answer given to the editor/reviewers. Response: We thank the reviewer for this question and apologise for not initially indicating the reason for this important change. In the initial manuscript, stability of our twenty putative reference genes was assessed across the seed coat and embryo tissues only. Upon revising the manuscript to include the source leaf, putative reference gene stability was assessed in the source leaf in addition to the seed coat and embryo. Utilising NormFinder, BestKeeper, RefFinder, and ắCt, we confirmed gene stability while geNorm revealed ALKBH6, HBP and IDE were the most effective combination of genes to be used as an internal reference for qPCR across the selected tissues of this study. 3. The following reference is still missing: Buttner 2010 The Arabidopsis sugar transporter (AtSTP) family: an update; Plant Biology doi:10.1111/j.1438-8677.2010.00383.x Response: The role of AtSTP13 in modulating plant susceptibility to pathogen infection has been investigated by Lemonnier et al., (2014) and Yamada et al., (2016), for this reason, these references have been added to the introduction. While Buttner (2010) elegantly summarised what was known about the Arabidopsis STP family at that point in time, we believe the references we have selected with respect to AtSTP10 and AtSTP13 are most appropriate. Reviewer #2: 1. The word "expressed" in the title denotes a gene which should be italicized. Otherwise, consider "present" or another descriptor to refer to the protein. Response: We agree with Reviewer 2 and have replaced “expressed” with “present”. 2. Methods - Plant Growth conditions, Course river sand should be "Coarse" river sand Response: We thank Reviewer 2 to for identifying this error and have replaced “Course” with “Coarse” 3. Please check the ordering of units in the following sentence as they are inconsistent: During light hours, plants were chronologically illuminated with 3h/200ʒPROVí Pí
Ví SKRWRV\QWKHWLFDFWLYHUDGLDWLRQ
3$5

Plant Direct
Open Access

K 3PROVí Pí 3$5DQG K 3mol Ví Pí 3$5 Response: We thank Reviewer 2 to for identifying this error and have revised the text to ensure consistency. 4. Please check italicisation of "MluI" in oocyte methods Response: We thank Reviewer 2 for identifying this oversight. The “u” has also been italicised to ensure the three letters referring to the species of origin is italicised. 5. Please consider revising "localisation to the source leaves" in the final paragraph as expression was measured rather than localization performed. Response: We agree with Reviewer 2 and have revised the text to remove “localisation” and appropriately refer to qPCR determination of PvSTP13.1 expression in the source leaf and seed coat. 6. Please remove hyphen after leaf in "hexoses from the leaf-" (unless there was meant to be one in seed coat-apoplasm) Response: A hyphen has also been included between coat and apoplasm. 7. Figure 6: Typo on panel labelling - "substarte" is used instead of "substrate" above panel D Response: We thank Reviewer 2 for identifying this error which has been fixed accordingly
